# Enhanced activity of highly conformal and layered tin sulfide (SnS_x_) prepared by atomic layer deposition (ALD) on 3D metal scaffold towards high performance supercapacitor electrode

**DOI:** 10.1038/s41598-019-46679-7

**Published:** 2019-07-15

**Authors:** Mohd Zahid Ansari, Nazish Parveen, Dip K. Nandi, Rahul Ramesh, Sajid Ali Ansari, Taehoon Cheon, Soo-Hyun Kim

**Affiliations:** 1 School of Materials Science and Engineering, Yeungnam University, Gyeongsan, 712-749 Republic of Korea; 2 Department of Chemistry, College of Science, King Faisal University, Al-Ahsa, Saudi Arabia; 3 Department of Physics, College of Science, King Faisal University, Al-Ahsa, Saudi Arabia; 4 Center for Core Research Facilities, Daegu Gyeongbuk Institute of Science & Technology, Sang-ri, Hyeonpung-myeon, Dalseong-gun, Daegu, 711-873 Republic of Korea

**Keywords:** Materials for energy and catalysis, Synthesis and processing

## Abstract

Layered Sn-based chalcogenides and heterostructures are widely used in batteries and photocatalysis, but its utilizations in a supercapacitor is limited by its structural instability and low conductivity. Here, SnS_x_ thin films are directly and conformally deposited on a three-dimensional (3D) Ni-foam (NF) substrate by atomic layer deposition (ALD), using tetrakis(dimethylamino)tin [TDMASn, ((CH_3_)_2_N)_4_Sn] and H_2_S that serves as an electrode for supercapacitor without any additional treatment. Two kinds of ALD-SnS_x_ films grown at 160 °C and 180 °C are investigated systematically by X-ray diffractometry, Raman spectroscopy, X-ray photoelectron spectroscopy, and transmission electron microscopy (TEM). All of the characterization results indicate that the films deposited at 160 °C and 180 °C predominantly consist of hexagonal structured-SnS_2_ and orthorhombic-SnS phases, respectively. Moreover, the high-resolution TEM analyses (HRTEM) reveals the (001) oriented polycrystalline hexagonal-SnS_2_ layered structure for the films grown at 160 °C. The double layer capacitance with the composite electrode of SnS_x_@NF grown at 160 °C is higher than that of SnS_x_@NF at 180 °C, while pseudocapacitive Faradaic reactions are evident for both SnS_x_@NF electrodes. The superior performance as an electrode is directly linked to the layered structure of SnS_2_. Further, the optimal thickness of ALD-SnS_x_ thin film is found to be 60 nm for the composite electrode of SnS_x_@NF grown at 160 °C by controlling the number of ALD cycles. The optimized SnS_x_@NF electrode delivers an areal capacitance of 805.5 mF/cm^2^ at a current density of 0.5 mA/cm^2^ and excellent cyclic stability over 5000 charge/discharge cycles.

## Introduction

The current demand for high energy and power density devices has encouraged the extensive research into new materials and techniques. The main focus is the development of electrochemical energy storage devices, especially in portable electronics and electric vehicles^1^. Among various devices, the supercapacitor, or electrochemical supercapacitor, is considered as a one of the prominent power sources and a good alternative to rechargeable batteries. The supercapacitor is unique as an energy storage device due to its high power density, fast charging/discharging rate, super long cycle life, and environmental friendliness^2,3^. Based on the energy storage mechanism, the supercapacitor can be classified as an electrostatic double-layer capacitor (EDLC), where the energy is stored by means of accumulating charge at the interface between the electrode and electrolyte. Alternatively, it can be categorized as a pseudocapacitor, where in addition to the EDLC, the energy is also derived partially from a Faradaic redox reaction with the electrode materials^4,5^. Generally, carbon-based materials work as double-layer capacitors, while the transition metal-based electrodes typically show pseudocapacitance behavior^6–9^. Transition metal dichalcogenides (TMDCs) of MX_2_, where M is the transition metal and X can be sulfur (S), selenium (Se), or tellurium (Te), are extensively studied in energy storage applications, due to their high capacity and high power density that could be attributed to their layered structures, high surface areas, and electronic conductivities^10–12^. Among them, the transition metal sulfides, such as molybdenum disulfide (MoS_2_) and tungsten disulfide (WS_2_) have been investigated mostly for improving the supercapacitor performance^7,13,14^.

Tin (Sn) -based chalcogenides, such as tin(II) sulfide (SnS), tin(IV) sulfide (SnS_2_), Sn_2_S_3_, S_3_S_4_, and tin selenide (SnSe), etc., also have various energy-related applications because of their robust structure, and outstanding electrical and optical characteristics^15,16^. Among them, SnS is found in an orthorhombic structure with each Sn atom bonded to six S chalcogen atoms, which forms a distorted octahedral geometry structure with an interlayer spacing of c = 0.433 nm (JCPDS No. 39-0345). In contrast, SnS_2_ occurs in a hexagonal unit cell where the central Sn atom is covalently bonded to six other S chalcogen atoms in the octahedral sites of individual layers. These layers consist of three atomic planes like the prototype of cadmium iodide (CdI_2_) with a larger interlayer spacing (c = 0.5899 nm, JCPDS No. 23-0677). This distinct layered structure with a bigger interlayer spacing could make it suitable for the insertion and extraction of guest species, make swelling tolerant hosting spaces, and increase the diffusion for guest ions including lithium cation (Li^+^), sodium cation (Na^+^), potassium cation (K^+^), hydrogen ion (H^+^), and hydroxide (OH^−^), etc. Owing to these unique crystallographic features, Sn chalcogenides are widely explored as active materials for lithium and sodium ion batteries and electrocatalytic applications^17–19^.

However, tin sulfides (SnS_x_) alone have not been widely explored as active materials for supercapacitor applications, since they suffer from relatively poor electrical conductivities and structural instabilities in electrochemical conditions, resulting in a limited cycling ability^7^. To solve the above problems, Sn-based composites or hetero-structured electrodes have been suggested with complex preparation procedures^20–26^. For example, Chauhan *et al*. and Wang *et.al*. synthesized SnS_2_/reduced graphene oxide (RGO) nanosheets and SnS_2_/MoS_2_ composited by the hydrothermal method and presented enhanced specific capacitances of 500 F/g and 220 F/g, respectively, with cycle stability up to 1000 cycles^22,24^. However, most of the studies involved complex and hybrid materials of SnS_x_ electrodes, rather than pure SnS_x_ with low specific capacitance and short cycle life^21–24^. To tackle the above issues, one promising approach is the direct and conformal growth of nanostructured transition metal sulfides on a three-dimensional (3D) conductive substrate. With this option, a porous substrate can provide a larger surface to volume ratio, which enables greater electrode/electrolyte contact. Besides, the composite electrode is binder-free, which can also contribute to enhanced electrochemical performance and an overall higher energy density. In this study, we suggest a composite of SnS_x_@3D Ni-foam (NF) as a promising electrode for a supercapacitor. For the direct and conformal growth of SnS_x_ films on 3D NF, atomic layer deposition (ALD) was adopted at relatively low temperatures below 200 °C. The major advantages of ALD over other deposition techniques such as evaporation, radio frequency (RF) sputtering, chemical vapour deposition (CVD), and spray pyrolysis, etc.^27–33^ are its ability to deposit highly uniform, thin film over a large surface. Furthermore, ALD allows for an extremely conformal coating of different materials with excellent thickness control, which is achieved by using sequential surface chemical processing and self-limiting reactions^34–41^.

ALD has been extensively researched in several energy-related fields that primarily include solar photovoltaic and secondary batteries (like Li/Na-ion, Li-S and Li-air batteries). To date, unfortunately, for supercapacitor electrode, very few studies have been done so far on direct active electrode materials. ALD-prepared VO_x_, TiN, TiO_2_, NiO, RuO_2_, Co_8_S_9_ and MoS_2_ are among few of them that have been previously investigated for supercapacitor electrode due to its high uniformity and conformality onto any desired multifaceted substrate^42–50^ (Table S1). For instance, Li et.al. developed ALD process to deposit Co_8_S_9_ on porous NF as a promising electrode for supercapacitor in terms of high specific capacitance, rate capability and long term cyclibility^47^. Similarly, our recent research work further established the potential of ALD by coating a uniform and conformal MoS_2_ nano-layer on 3D NF, exhibited noteworthy performance for supercapacitor electrode^13^. Therefore, these studies truly suggest the potential of this method in developing the thin films of metal sulfides for direct fabrication of supercapacitor electrodes without any additional treatment. In this study, we prepared SnS or SnS_2_ predominant SnS_x_ films by controlling the deposition temperature^35,39,40^ in order to comparatively investigate their supercapacitor’s performances. For the first time, two kinds of ALD-SnS_x_ films were directly grown on NF, and then the composites of ALD-SnS_x_@NF were tested as active electrodes without any additional treatment. The coverage of free-standing 3D NF surface by ALD-SnS_x_ provides superior electric connection and enhance the overall capacitance as a result of additional Faradaic reaction. The fundamental layer-by-layer features of ALD can protect the SnS_x_ film from decomposition, deformation and depletion in the long-term cycling performance test.

## Results and Discussion

### Physical characterizations of ALD-SnS_x_ films with deposition temperature

#### Phases characterized by XRD, Raman spectroscopy, and XPS

**Figure 1:**
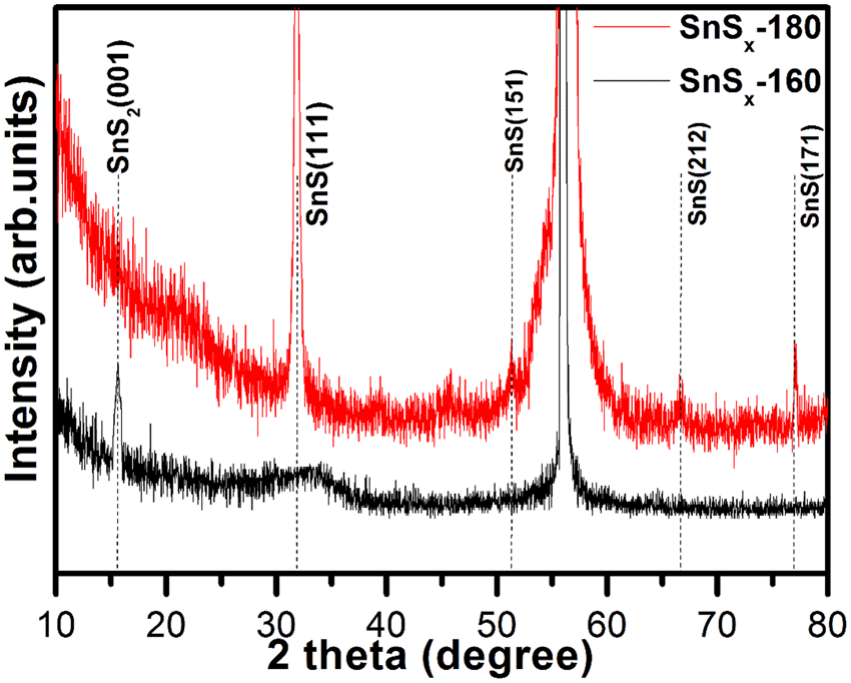
GIAXRD patterns of the as-grown ALD-SnS_x_ films on Si/SiO_2_ substrate.

The crystallographic structure and phase of the as-grown SnS_x_ films deposited on Si/SiO_2_ substrates at two different temperatures of 180 °C and 160 °C (denoted as SnS_x_-180 and SnS_x_-160) were studied using the grazing incident angle XRD (GIAXRD). The XRD patterns of SnS_x_-180 showed sharp peaks consistent with the orthorhombic structure of SnS (JCPDS No. 39-0354) with the space group of *Pnma* (space group 62), and the peak of the (111) crystallographic plane at reflection 2θ = 31.8° was far more intense than others (Fig. 1). Conversely, the XRD results for the SnS_x_-160 sample exhibited diffraction patterns conforming to the phase of hexagonal SnS_2_, and one clear peak was observed at approximately 2θ = 15.7°, corresponding to the (001) crystallographic plane (JCPDS No. 23-0677) with the space group of *P-3m1* (space group 164). However, a broad hump centred at 2θ around 32.7°, corresponding to the (111) crystallographic plane of SnS, was also observed. From the XRD patterns of SnS_x_-160, it can be inferred that thin film is composed of crystalline SnS_2_ and amorphous SnS. The lattice parameters are a = 4.13, b = 11.41, and c = 4.90 Å for the orthorhombic SnS (SnS_x_-180) and a = 3.55, b = 3.51, and c = 5.82 Å for hexagonal SnS_2_ (SnS_x_-160) calculated from the least square fitting to the Bragg peaks. Table S2 summarizes the average crystallite size, d-spacing determined from the XRD data by the Scherrer formula and X’Pert High score software. As shown in Table S2, the crystallite size of SnS increased as the deposition temperature increased to 180 °C. The shape of crystalline grain is not exactly spherical; therefore, the calculated values from the Scherrer formula only present a rough estimation for comparison. The interlayer spacing (d-spacing) of SnS grown at 180 and 160 °C corresponding to the strongest XRD peak at 2θ = 31.8 and 15.7°, is found to be 0.28 and 0.59 nm respectively. Previous studies^35^ have also demonstrated that SnS film is predominantly formed above 180 °C, whereas the single phase of SnS_2_ film is formed at a slightly lower deposition temperature of 160 °C. These findings suggest that the formation of SnS is more favourable at high temperatures, as compared to relatively low temperatures. From these observations and others, it is clear that temperature plays a crucial role in determining the phase of ALD-grown thin film^12,34–36^.

**Figure 2:**
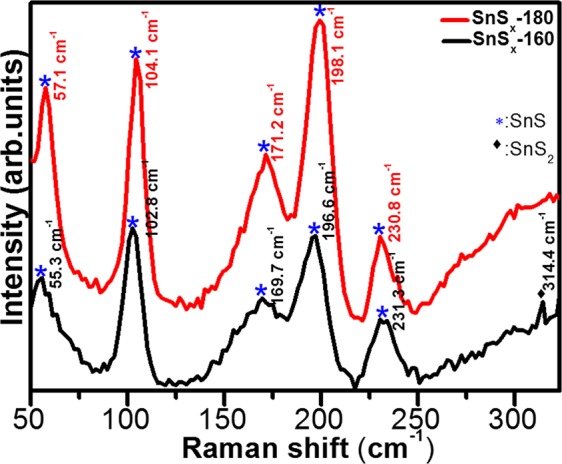
Raman spectroscopy spectra of the as-grown ALD-SnS_x_ thin films on Si/SiO_2_substrate.

Interestingly, for the XRD patterns of SnS_x_-180 deposited on NF, a diffuse peak from orthorhombic SnS (111) was observed (Fig. S1a); however, a diffraction peak from SnS_2_ was not detected for the SnS_x_-160 deposited on NF. This is attributed to the existence of sharp Ni peaks and low mass content of the active materials^13^. In addition, the crystalline growth of the film on Ni-foam was not completely discarded, which is presented and discussed in the following sections in this article. In order to gain more insight into the phase of SnS_x_, Raman spectra, which delivers a structural fingerprint of molecules, were recorded and are shown in Figs 2 and S1b. For comparison, we used reference data from a single crystal of SnS_2_ and SnS because Raman active modes of polycrystalline thin films can be complex, due to the shifting and broadening of the peaks. This is attributed to the grain boundaries, stretched defects, and stresses in the polycrystalline thin films. The Raman spectra of all the SnS_x_ samples showed distinguishable peaks (second to fifth) located at, 102 ± 3 cm^−1^ (A_g_), 169 ± 2 cm^−1^ (B_3g_), 196 ± 2 cm^−1^ (B_2g_), and 230 ± 2 cm^−1^ (A_g_), which correspond to first order single phonon oriented transverse optical (TO) and longitudinal optical (LO) vibrational modes of SnS. The first Raman peak at 56 ± 2 cm^−1^ [B_2g_(LO)-A_g_(TO)] and last peak at approximately 313.4 cm^−1^ [A_g_(TO) + A_g_(LO2)], can be assigned to the second order multiple phonons scattering process. In addition, the observed Raman active peak at 56 ± 2 cm^−1^ belongs to the vibrational mode of SnS phase, whereas the specific Raman peak at 313.4 cm^−1^ (only can be seen in the SnS_x_-160 sample) is associated with optical phonon mode of 2H-SnS_2_ poly-type with a hexagonal symmetry, and is related to Sn-S bonding in the a-c plane^18,35^. Moreover, the Raman spectra obtained from SnS_x_@NF (Fig. S1b) reflects further the phase dependent growth of SnS and SnS_2_ dominant phase at different temperature. The Raman spectra of the SnS_x_ film grown at 160 °C clearly exhibited a broad and strong peak at around 318 cm^−1^ that is completely absent when the film is grown at 180 °C, matches well with Raman mode of SnS_2_ phase. This observation was in agreement with the XRD results of the SnS_x_ film grown at 160 °C, which showed the formation of a hexagonal SnS_2_ phase. Furthermore, as compared to previous Raman studies, a significant red-shift in the position of all the Raman peaks has been detected^51–53^. The slight shifts in peaks are also consistent with those previously reported for SnS_x_ thin film deposited particularly at higher temperatures. From this study and others, it could be expected that the change in peak position in the Raman spectra mainly relies on their exterior surface texture or roughness^51–53^. A previous study on electrodeposited SnS by Mathew *et al*. revealed traces of other phases by Raman spectroscopy^54^.

**Figure 3:**
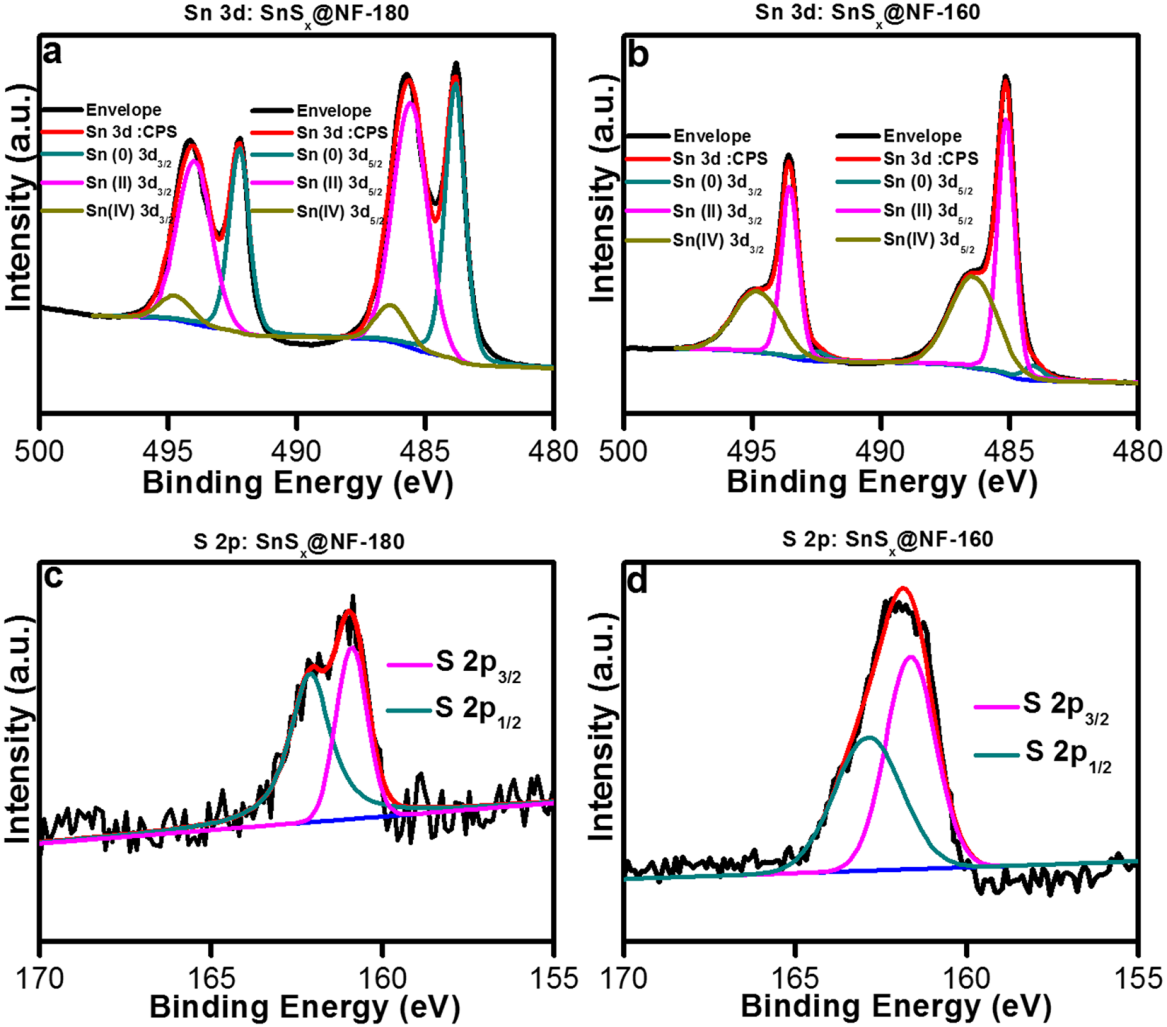
X-ray photoelectron spectroscopy spectra of Sn 3d region for (**a**) SnS_x_@NF-180 and (**b**) SnS_x_@NF-160; S 2p region for (**c**) SnS_x_@NF-180 and (**d**) SnS_x_@NF-160.

In order to investigate the oxidation state, chemical composition, and constituent elements of as-grown SnS_x_ films, X-ray photoelectron spectroscopy (XPS) measurements were carried out. The contaminations and various oxidation states present on the active materials play a crucial role in determining their fundamental properties, which can affect the electrochemical reactions^17^. It is well-known that Sn chalcogenides have versatile oxidation characteristics, and focusing on their compositions can clarify their electrochemistry. Argon ion sputtering at the surface of the samples was performed for 60 s prior to the XPS measurement to remove unwanted surface species, such as oxides and other contaminants, from the SnS_x_ films. Inevitably, surface oxidation of both ALD-grown SnS_x_ films revealed their oxygen content to be around 10.7% in SnS_x_-180 and 7.3% in SnS_x_-160 film by a full survey of XPS analysis (Fig. S2 & Table S3). In this analysis, the Sn 3d and S 2p core level XPS spectra of SnS_x_@NF-180 and SnS_x_@NF-160 displayed clear differences (Figs 3 & S3). The binding energy (BE) values were calibrated based on the adventitious carbon 1 s peak (284.8 eV). The oxidation states of Sn in SnS_x_ were established from the deconvoluted XPS spectra of the SnS_x_@NF-180 (Fig. 3a). The 3d_5/2_ transition was deconvoluted into three peaks at BE values 483.8 eV, 485.6 eV, and 486.5 eV, corresponding to the Sn oxidation state of 0, + 2 and + 4, respectively^11,12^. The XPS analysis suggests that the dominant phase is SnS for the thin film deposited at 180 °C; however, the formation of metallic Sn in notable quantities was also evident.

Similarly, the SnS_x_@NF-160 sample also showed a pair of doublets at BEs (3d_5/2_) of approximately 485.2 eV and 486.4 for Sn(II) and Sn(IV) states, respectively (Fig. 3b). The XPS analysis of samples grown at a 160 °C showed an increase in the peak intensity from a Sn oxidation state of +4 and the disappearance of Sn oxidation state of 0, which suggests the formation of a mixed phase of SnS and SnS_2_. The sulfur 2p core-level XPS spectrum from SnS_x_@NF-180 and SnS_x_@NF-160 are shown in Fig. 3c,d. The spectrum of SnS_x_@NF-180 sample showed two peaks at BEs of approximately 160.8 eV and 162.1 eV, corresponding to the S 2 p_3/2_ and S 2p_1/2_ electronic states, respectively. Whereas for the SnS_x_@NF-160 sample, these peaks appeared at slightly higher BEs of 161.7 eV (S 2p_3/2_) and 162.9 eV (S 2p_1/2_). The shift in peak position is attributed to the change in the stoichiometry of the film. Thus, the XPS results also confirmed that the dominant phase of the film deposited at 180 °C is SnS (Sn^2+^) and that at 160 °C is SnS_2_ (Sn^4+^)^17,40,41^. Atomic weight percentages of the deconvoluted states of Sn are recorded in Table S4 from the core-level spectra using the CASA XPS software. The atomic percentages of the various valence states in SnS_x_-180 and SnS_x_-160 films were calculated and showed that Sn elements with valence state of + 2 and +4 are dominant species in SnS_x_-180 and SnS_x_-160 film, respectively, which agrees with the phase analyses by XRD. Owing to the various oxidation states of Sn (Sn^+4^, Sn^+2^, and Sn^0^), thermodynamically stable Sn oxides have often been found as SnO or SnO_2_ on the surface of SnS and SnS_2_^55^.

#### Morphologies and microstructures characterized by SEM and TEM

**Figure 4:**
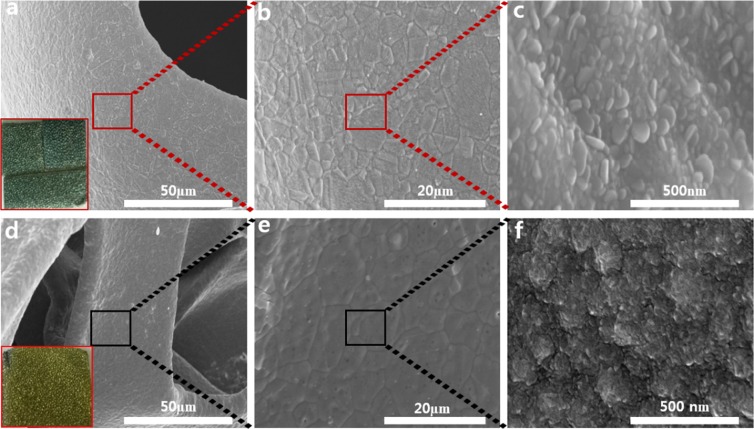
SEM images of (**a**–**c**) SnS_x_-180 and (**d**–**f**) SnS_x_-160 grown on 3D NF by 500 ALD cycles (Inset: digital photograph of SnS_x_@NF at 180 °C and 160 °C).

Scanning electron microscopy (SEM) images were recorded to recognize morphological changes in ALD-SnS_x_ thin films grown at two different deposition temperatures. Figure 4 displays the surface morphology of SnS_x_ films grown at deposition temperatures of 180 °C and 160 °C on an NF substrate. It was evident that the prepared SnS_x_ films grew uniformly on the entire NF surface at both temperatures. This highlights the potential of ALD towards conformal and uniform coating on a complex structure such as NF. The SnS_x_@NF-180 film showed the formation of flakes type morphology (Fig. 4c), whereas the SnS_x_@NF-160 film exhibited large granules-like morphology with relatively higher surface roughness, resulting in higher interface area between the electrode and electrolyte during charge storage (Fig. 4f). This indicates that surface morphology can be modified by simply adjusting the deposition temperature. Sinsermsuksakul *et. al*. and Ham *et.al*. reported similar behaviour on ALD-SnS_x_ films with deposition temperature^36,40^. Moreover, from digital photographic images, SnS and SnS_2_ predominant ALD-SnS_x_ films were clearly noticeable from their exterior, where SnS is grey and SnS_2_ is golden yellow^17^. Further, an elemental analysis of SnS and SnS_2_ were performed using EDS (Fig. S4) where the acquired chalcogen to the metal ratio for SnS_x_@NF-180 is ~0.87 and 1.7 for SnS_x_@NF-160 (Table S5). In both cases, the estimated ratios clearly indicated the predominant formation of SnS and SnS_2_ phase at two different deposition temperatures. Chalcogen to metal ratios obtained from the XPS analysis was also in line with these EDS results. Therefore, ALD-grown SnS_x_ films at 180 °C (SnS rich-SnS) and 160 °C (SnS_2_ rich-SnS) have been successfully synthesized with distinct difference in phase and stoichiometry by changing the deposition temperature. Figure S5 shows the corresponding energy dispersive X-ray (EDS) elemental distribution mapping for the SnS_x_ film on the 3D NF. It is confirmed a uniform distribution of Sn and S in the deposited thin film on 3D NF. It is difficult to achieve conformal coating on the NF with extreme precision of the film thickness by any other material synthesis technique. Therefore, ALD provides us with a direct and easy fabrication of an NF-supported composite electrode for efficient supercapacitor application.

**Figure 5:**
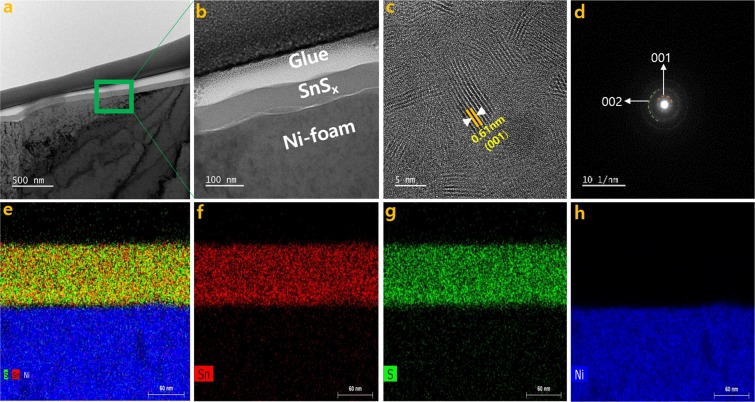
(**a,b**) Cross-sectional view bright-field TEM images, (**c**) the HRTEM images, (**d**) SAED patterns for the SnS_x_@NF-160 grown by 500 ALD cycles, and (**e**–**h**) the corresponding STEM elemental mapping confirming the uniform distribution of Sn, and S, on Ni foam.

The TEM analysis for SnS_x_@NF-160 was carried out to characterize the phase and microstructure of SnS_x_ thin film in detail. The images (Fig. 5a–c), selected area electron diffraction (SAED) patterns (Fig. 5d), and the EDS elemental mapping (Fig. 5e–h) were obtained. The TEM images confirmed the conformal coating of Ni-foam substrate with 60 nm of SnS_x_ film (Fig. S6). The high-resolution TEM images (Fig. 5c) clearly demonstrated a polycrystalline layered structure of SnS_2_ film with an inter-layer spacing of approximately 0.61 nm. The lattice fringes can be indexed to the (001) plane of the hexagonal SnS_2_, where the Sn atoms are sandwiched between two layers of hexagonally close-packed S atoms, while the adjacent sulfur layers are connected by the weak van der Waals interaction^12,40^. Layered structured film could provide an increase in surface area and a greater number of accessible active sites, which would allow for improved electrode/electrolyte contact and enhanced charge storage capacity^14^. The layered nature could also provide effective channels for the proper mass transport of electrolyte ions within the electroactive material, thereby causing speedy redox reactions and charge adsorption on the electrode surface^14,56^. The polycrystalline nature of the materials was further confirmed by the SAED patterns (Fig. 5d). The observed diffraction rings were from the (002), and (001) planes of the hexagonal SnS_2_ structure. Hence, the TEM diffraction pattern results are well matched with the XRD results. Once again, the STEM-EDS elemental mapping (Fig. 5e,f) of the SnS_x_@NF-160 sample confirmed the uniform distribution of elemental Sn (Fig. 5f) and S (Fig. 5g) on Ni-foam.

### Electrochemical characterizations of supercapacitor

#### Phase-dependent studies

**Figure 6:**
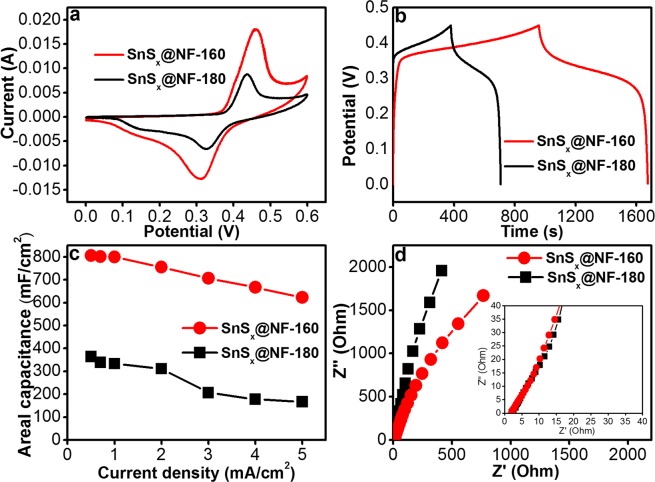
(**a**) Comparative CV curves at 10 mV/s scan rate, (**b**) Galvanostatic charge-discharge (GCD) curves at current density of 0.5 mA/cm^2^, (**c**) Areal capacitances as a function of current density, and (**d**) Electrochemical impedance spectroscopy (EIS) for ALD-SnS_x_@NF-160 and ALD-SnS_x_@NF-180 at a fixed thickness of 60 nm.

In order to investigate its performance as an electrode for a supercapacitor, the electrochemical characterizations of the SnS_x_@NF electrodes were performed in a three-electrode system. While 2M KOH aqueous solution was used as the electrolyte, standard Pt and Ag/AgCl electrodes were applied as the counter and reference electrodes, respectively. The supercapacitor performance of SnS_x_@NF-180 and SnS_x_@NF-160 at fixed 60 nm was thoroughly investigated with cyclic voltammetry (CV), galvanostatic charge/discharge (GCD), and electrochemical impedance spectroscopy (EIS) measurements. By adjusting the number of ALD cycles, the thickness of the active electrodes was fixed at 60 nm for both SnS_x_@NF-180 (670 ALD cycle) and SnS_x_@NF-160 (500 ALD cycle). Figure 6a shows CV curves for the SnS_x_@NF-180 and SnS_x_@NF-160 electrodes at a 10 mV/s scan rate within a potential window of 0–0.6 V. As can be seen from the figure, the CV curves of these electrodes clearly depicted both electrical double layer capacitance (EDLC) and Faradaic characteristics, which renders this composite an efficient electrode for a supercapacitor^13,57^. It was also evident that the CV curve of SnS_x_@NF-160 exhibited a larger area than that of SnS_x_@NF-180, presenting the former as a superior supercapacitor electrode material as compared to the latter. This enhanced performance of the SnS_x_@NF-160 electrode is mainly attributed to the polycrystalline-layered structure of SnS_2_, which facilitated the rapid transport of the electrolyte ions, and thus enhanced both the EDLC and the Faradaic contribution for this electrode^14,58–62^. Figure 6b presents charge/discharge curves of SnS_x_@NF grown at 180 °C and 160 °C, at a current density of 0.5 mA/cm^2^ within a potential window of 0–0.6 V used for CV measurements. The charge/discharge times of the SnS_x_@NF-160 and SnS_x_@NF-180 electrodes were 1676 s and 717 s, respectively. The higher charge, as well as discharge time for the SnS_x_@NF-160 electrode, reconfirms its enhanced charge storage ability. The areal capacitance was calculated from the charge/discharge curves of the SnS_x_@NF-180 and SnS_x_@NF-160 electrodes at different current densities (Fig. 6c). The areal capacity of individual SnS_x_@NF composite electrodes decreased with an increase in operational current density. This is due to the lower absorption/desorption or intercalation of electrolyte ions into the electrode at higher current densities. The inner active sites may not have taken part in the redox reaction, possibly due to a lower diffusion of ions within the electrode and the positive K^+^ ions only reaching the outer surface of the electrode materials. With the increase in the current rate from 0.5 mA/cm^2^ to 5 mA/cm^2^, the areal capacitance was decreased considerably from 805.55 mF/cm^2^ to 622.22 mF/cm^2^ in the case of SnS_x_@NF-160, and from 364.44 mF/cm^2^ to 166.66 mF/cm^2^ for SnS_x_@NF-180. In particular, at a current density of 0.5 mA/cm^2^, the SnS_x_@NF-160 composite revealed a higher areal capacitance of 805.55 mF/cm^2^ than that of the SnS_x_@NF-180 electrode (364.44 mF/cm^2^), due to its unique layered structure. The results obtained with the SnS_x_@NF-160 composites are also better than several previous reports with other electrode materials (Table S8) as well as comparable with the areal capacitance reported earlier for ALD-grown electrodes used in supercapacitor (Table S1). These enhanced performances could be attributed mainly to the uniform and conformal coverage of SnS_x_ film on high-surface-area NF that exhibited a higher concentration of active sites. It also enables a better electric contact with the unique layered structure, providing large interlayer spacing for the easy migration of the electrolyte ions into the electrode.

To further understand the dynamics of the interfacial charges and their transfer process between the electrode and electrolyte, electrochemical impedance spectroscopy (EIS) measurements were performed at a frequency range of 1 Hz to 50 kHz. Figure 6d presents Nyquist plots for the SnS_x_@NF-180 and SnS_x_@NF-160 electrodes. The typical characteristics of a supercapacitor were revealed from these Nyquist plots. It can be seen that the shape of impedance spectra from two electrodes are similar, which consist of a quasi-semicircle in the high frequency region and a straight oblique in the low frequency region. After a close inspection, the magnified region in the inset of Fig. 6d confirmed that the Nyquist plot contains slightly different characteristics. At the high-frequency region, a tilted straight line is observed (diffusion behavior) instead of a semicircle which is attributed to the transport limitation of K^+^ ion through the distributed resistance-capacitance in the porous nickel foam substrate. Generally, solution resistance of the electrolyte (Rs) denotes the internal resistance and the numerical values of real-axis intercept in the high-frequency range can be applied to evaluate its size. The diameter of the other semicircle at the low-frequency region belongs to charge transfer impedance due to the pseudocapacitive behavior. Whereas the slope of the straight line representing the Warburg impedance, which might be attributed to the electrolyte ion diffusive impedance and proton diffusion inside the SnS_x_ electrode^13^. As observed in the figure, the high frequency region reflects the combined charge transfer resistance (Rct) and double layer capacitance at the electrode-electrolyte interface. Further, at the low-frequency region, Warburg impedance can be seen from the straight oblique, which indicates the electrolyte ion or proton diffusion within the electrode. The Warburg resistance (diffusion resistance) of the SnS_x_@NF-180 and SnS_x_@NF-160 electrode is found to be 0.36 and 0.20 ohm/s, respectively. Inferior values of resistance are beneficial for improving the storage capacity of the electrode materials. These results indicate that the lower Warburg resistance and larger interlayer spacing of layered SnS_2_ in the SnS_x_@NF-160 composite might be responsible for improving the diffusion of ionic charge carriers. Furthermore, the diffusion coefficient D_0_ for the prepared electrode in KOH medium was calculated by applying the Randles-Sevcik equation^63^,

1 $$Ip=(2.687\times {10}^{5}){n}^{3/2}AC{{D}_{o}}^{1/2}{v}^{1/2}$$

where *I*_*p*_ is the peak current position, *n* is the number of electrons transferred in the redox reaction, *A* is the effective electrode area of the working electrode in cm^2^, *C* is the concentration of the diffusing species in the bulk of the electrolyte, and *v* is the voltage scan rate. According to the above equation, the diffusion coefficient of electrolyte ions at the interfacial region is calculated to be 5.44 × 10^−11^ and 2.27 × 10^−10^ cm^2^/s for the SnS_x_@NF-180 and SnS_x_@NF-160 electrode, respectively. It is believed that the enhanced values of the SnS_x_@NF-160 composite are caused by the layered structure with the larger interlayer spacing provides a larger surface area and more active sites for the ion transport. Therefore, the enhanced performance of SnS_x_@NF-160 electrode further confirmed the optimum electrode for achieving the better performance in this study.

#### Thickness-dependent studies

From these analyses, it is clear that SnS_x_@NF-160 is the better choice for this study because it contains a predominant phase of Sn (IV) with some quantities of Sn (II), which confirms the formation of SnS_2_ with its well-known hexagonal layered structure and higher intrinsic conductivity contribution from the SnS phase. For this particular study, it can be concluded that the SnS_2_ phase, which was predominant in SnS_x_@NF-160, performs better as an electrode in a supercapacitor. Furthermore, the thickness of the active electrode material used in an energy storage device also plays a critical role. The performance of such a storage device is subjected to degrading beyond a certain thickness of the active electrode layer. An unnecessary mass loading beyond a critical thickness may severely affect the capacitance of the device. This is caused by two different factors acting simultaneously with each other. The extra thickness, which does not take part in the electrochemical process of storing the charge, will at the same time provide some extra electrical resistivity to the whole electrode. In this regard, ALD can be considered as one of the best techniques with its precise control over film thickness. The following sections of this article present an optimized thickness for SnS_x_@NF-160 to maximize the performance of the supercapacitor, by controlling the number of ALD cycles.

**Figure 7:**
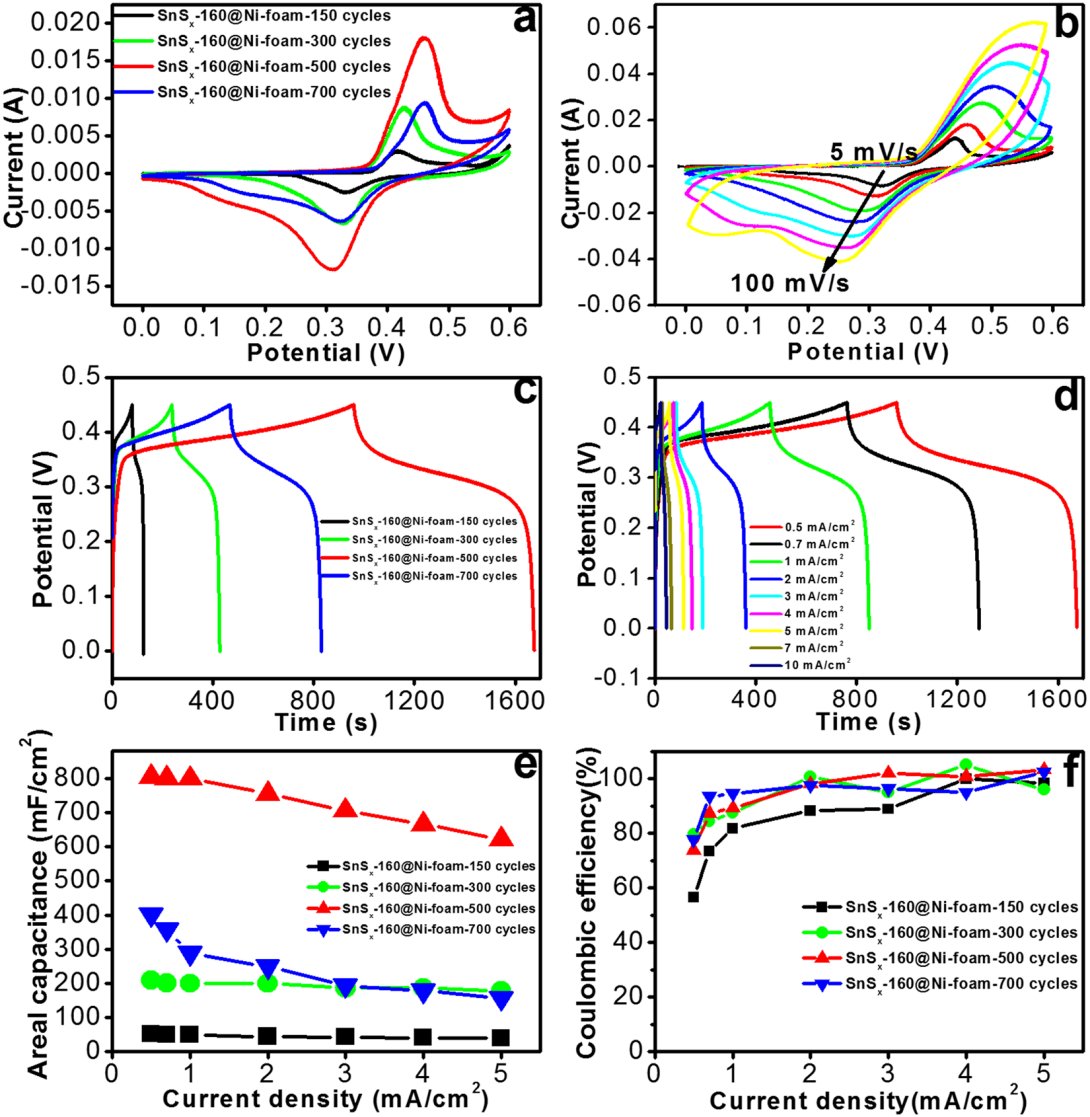
(**a**) CV plots of ALD-SnS_x_@NF-160 grown by 150, 300, 500, and 700 ALD cycles at a scan rate of 5 mV/s, (**b**) CV curves at different scan rates for the ALD-SnS_x_@NF-160 electrode grown by 500 ALD cycles, (**c**) Charge/discharge profiles at a current density of 3 mA/cm^2^ for the above four ALD-SnS_x_@NF-160 electrodes, (**d**) Charge/discharge profiles at different current densities for ALD-SnS_x_@NF-160 grown by 500 ALD cycles, (**e**) Areal capacitances at different current densities for the three different electrodes, and (**f**) their corresponding Coulombic efficiencies (CEs).

The CVs of SnS_x_@NF-160 prepared with the different ALD cycles and thicknesses [150 (18 nm), 300 (36 nm), 500 (60 nm) and 700 (84 nm)] are shown in Fig. 7a. The CV results suggest the presence of pseudocapacitance behaviour with apparent redox peaks. Two types of pseudo-capacitive behaviours were evident: (1) a broad peak in the central region similar to that observed in a battery like Faradaic reaction and (2) broad diffuse Faradaic processes, which was likely from the composite materials. This complex behaviour is because of the combined effect of double layer capacitance (non-faradaic) and pseudocapacitance (Faradaic) processes, possibly due to the insertion/extraction of K^+^ from the interlayer of the SnS_2_^7,13,57^. To obtain further information on the effect of voltage scan rates on the capacitive response of the electrodes, the CV results were recorded at different scan rates (Fig. 7b) for the SnS_x_@NF-160 sample (500 ALD cycle) with the best performance. The area under the redox peaked and EDLC increased with the scan rate, while the anodic (discharge) peak shifted towards higher voltages and the cathodic (charging) peak shifted to lower values^57^. The increase in the current response is an indication of superior kinetics and better reversibility of the interfacial Faradaic redox reactions and the fast rate of electronic or ionic transport. Similar features were also observed in CVs and the charge/discharge curves of all the electrodes prepared with different ALD cycles (Figs S7 & S8a).

To further examine the electrochemical behaviour of the SnS_x_@NF-160 electrodes, GCD measurements were carried out at different numbers of ALD cycles by maintaining a potential window of 0–0.45 V (Fig. 7c). The charge/discharge time period (total duration for a complete charging and discharging cycle) for samples prepared with 150 ALD cycle was approximately 124 s and extended to 1676 s for samples prepared with 500 ALD cycles. This signifies an increase in capacitance with the thickness of SnS_x_ thin film. However, further increases in ALD cycle numbers (700 ALD cycles, thicker film) and a drastic drop in charge/discharge time period (approximately 832 s) were also detected. These findings correspond with the results from the CV measurements. The decrease in capacitance with thickness (150, 300, and 700 ALD cycles) beyond an optimal value of approximately 500 ALD cycles may be due to the blocking of electrolyte ions (K^+^ and OH^−^) and higher electrode resistance (Fig. S9). Unlike the linear charge/discharge profiles generally reported for pure EDLC, the charge/discharge profile of the SnS_x_@NF composite electrode appeared to plateau between 0.24 V and 0.34 V (discharge), which is attributed to the Faradaic reactions. The charge/discharge profiles were symmetrical except for a slight curvature, indicating the contribution of a pseudocapacitive process along with the electric double layer capacitance^10,20^. The charge/discharge profiles of SnS_x_@NF-160 grown with 500 ALD cycles were measured at different current ranges of 0.5 mA to 10 mA (Fig. 7d). The curves were also symmetrical except for a slight curvature, which points out the pseudocapacitive contributions towards the total capacitance. The areal capacity estimated from the charge/discharge profile of SnS_x_@NF-160 prepared with different ALD cycles (Fig. 7c) is shown in Fig. 7e. The highest areal capacitance (e.g. 805.55 mF/cm^2^ at 0.5 mA/cm^2^ current density) was observed with the SnS_x_ thin film grown by an optimal ALD cycle number of 500. A further increase in the SnS_x_ ALD cycles (700 ALD cycles) led to a drastic decline in the capacitance (402 mF/cm^2^ at 0.5 mA/cm^2^). Additionally, the areal capacitance gradually decreased with an increase in the operational current density, and the samples prepared with more ALD cycles demonstrated steeper drops. The charge/discharge curves at different current densities for all SnS_x_ electrodes (150, 300, and 700 ALD cycles) are shown in Figs S10 & S8b and a gradual decrease in the discharge time with increasing current density was evident. The reason for this sudden fall in capacitance can be explained based on two factors. The first is the low penetration of the electrolyte ions into the bulk of the deposited SnS_x_ film above a certain critical thickness, and the second is the increase in electrode resistivity with thickness. A thicker SnS_x_ film with a relatively high resistivity will impede the transport of both electrons from the NF and ions from the electrolyte^13^. The CE of all SnS_x_@NF-160 composite electrodes at different current densities (0.5–5 mA/cm^2^) was calculated from the ratio of discharge to the corresponding charge time and the values are shown in Fig. 7f. At higher current density (1–5 mA/cm^2^), the optimized SnS_x_@NF electrode grown with 500 ALD cycles presented CE values near to 100%. However, a lower CE was recorded (slightly higher than 80%) for this electrode at low current densities of 0.5 mA/cm^2^ and 0.7 mA/cm^2^, which could be attributed to the higher ratio of side reactions at low current density than that at high current density. A lower CE was achieved for the other three SnS_x_@NF-160 electrodes with 150, 300, and 700 ALD cycles, which is consistent with the other inspections above.

It is well known that the total electrochemical charge stored in the electrode can be separated by the diffusion-controlled faradaic process and capacitive process. The kinetic analysis is performed to differentiate the diffusion-controlled capacity from the capacitive one. Generally, the faradaic process further includes the diffusion controlled behavior from the conversion/alloying reaction and redox pseudocapacitive effects from the charge transfer with surface/subsurface atoms. The individual specific contribution from the diffusion controlled and capacitive processes could be obtained using the following equation^64^,

2 $$i({\rm{V}})={k}_{1}(v)+{k}_{2}({v}^{1/2})$$

where *i* is the current in CV curves at a fixed potential (V), *v* is the scan rate, the term *k*_1_(*v*) and *k*_2_(*v*^1/2^) represents the current response from the surface capacitance and diffusion-controlled faradaic process, which can be calculated by analyzing CV current at various scan rate.

To linearize, both sides of the Eq. (2) are divided by square root of the scan rate,

3 $$i({\rm{V}})/{v}^{1/2}={k}_{1}{v}^{1/2}+{k}_{2}$$

**Figure 8:**
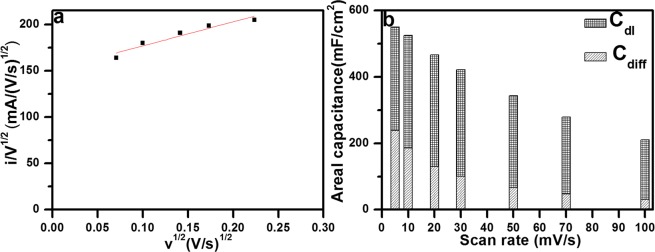
(**a**) By Eq. (1) to analyze the voltammetric sweep data for the SnS_x_@NF-160 electrode sweep rates varied from 0.5 to 10 mV/s, and (**b**) Comparative total capacity contribution obtained of capacitive and intercalation specific capacitance as a function of scan rate.

Now, the values of *k*_1_ and *k*_2_ can be determined by plotting *i*/*v*^1/2^ vs. *v*^1/2^ from the slope and intercept of the linear fit of the plot as shown in Fig. 8a. Thus, the current contribution through the capacitive processes and diffusion controlled (K^+^ intercalation/deintercalation) can be easily distinguished quantitatively at each potential for a fixed scan rate. The contribution of capacitive effect and diffusion controlled faradaic process in total stored charge of the SnS_x_@NF-160 electrode at various potentials is presented in Figs 8b and S11a. It can be seen that the double layer charging is slightly larger than diffusion controlled, meaning a significant proportion of the capacitance is resulted from the insertion/deinsertion mechanism, specifically at a low scan rate, like 56.46% at 5 mV/s. The contribution from the double layer charging process was increased to 64.71, 72.17, 76.05, 80.39, 82.91 and 85.29% as the scan rate increased 10 to 100 mV/s, suggesting the significant role for capacitive charge storage in the total capacity of the electrode. On the other hand, as the scan rate increased from 5 to 100 mV/s, the capacitance contribution from diffusion-controlled mechanism gradually decreases from 43.53–14.70%. Therefore, with increasing scan rate, the capacitive current increased gradually and intercalation current (diffusion-controlled) decreases and vice versa. It should be noted that at high scan rates, the electrolyte ions do not have enough time to insertion/extraction into/from the SnS_x_ layer. Thus, the capacitive behavior at the surface of the electrodes become dominant which delivers fast charge/discharge characteristics compared to the slow ion intercalation/deintercalation kinetics owing to the diffusion-controlled process^65^. At higher scan rates, the electrolyte ions mainly undergo adsorption/desorption at the electrode/electrolyte interface.

Furthermore, the amount of areal capacity contribution from the inner and outer surface of the optimized electrode (SnS_x_@NF-160, 500 ALD cycles) is calculated another method using Trasatti analysis. According to Trasatti theory, the maximum total capacitance can be determined as the sum of the capacitance provided by the inner and outer surface of the electrode materials, which can be expressed using the following equation^66^,

4 $${{\rm{C}}}_{total}={{\rm{C}}}_{in}+{{\rm{C}}}_{out}$$

Areal capacitances at different scan rates were calculated and plotted versus the square root of the scan rates (*v*^1/2^) as well as the inverse square root (*v*^−1/2^) of the same to determine those two contributions separately. The charge stored from the insertion process and the outer surface of the electrode is mainly dependent upon the specific scan rate (*v*). In this plotting (Fig. S11b), the maximum areal capacity for this electrode is calculated by extrapolation of capacitance value at *v* = 0 (intercept of the fitted straight line) from the plot of the areal capacitance (C_areal_) *vs*. square root of the scan rate (*v*^1/2^), since the diffusion of electrolyte ions into the electrode is maximum when the scan rate tends to zero. On the other hand, the areal capacitance contributed by the outer surface is found from the intercept (*v* = ∞) of C_areal_ versus reciprocal of the square root of scan rate (*v*^−1/2^) in the Fig. S11c where the diffusion of electrolyte ions into the electrode is supposed to be absent or negligible. Once we obtained the above two values, the capacitance contribution of the inner part of the electrode can be calculated. The SnS_x_ electrode grown by 500 ALD cycles presented a total capacitance of ~682 mF/cm^2^ and the capacitance exhibited by the outer surface was ~304 mF/cm^2^. Thus, the capacitance contribution exhibited by the inner surface of the electrode is ~378 mF/cm^2^; indicating most of the capacitance was contributed by the inner surface regions of the electrode. It thus can be said, more numbers of active sites are present in the inner surface region of the electrode and the smooth intercalation of ions during the electrochemical process should be significantly facilitated by the layered structure of SnS_2_.

#### Cyclic stability test

**Figure 9:**
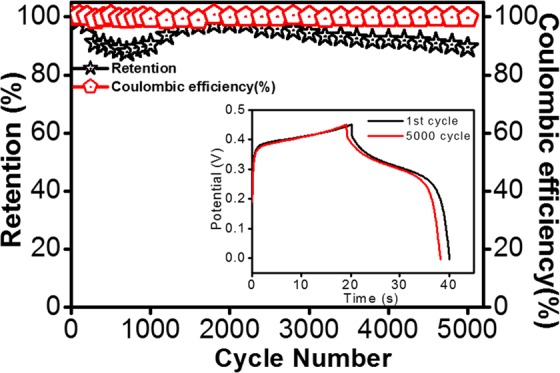
Cyclic stability as shown by the capacitance retention and the corresponding CE for ALD-SnS_x_@NF-160 grown by 500 ALD cycles. Inset figure shows charge/discharge curves of the 1^st^ and 5000^th^ cycles.

**Figure 10:**
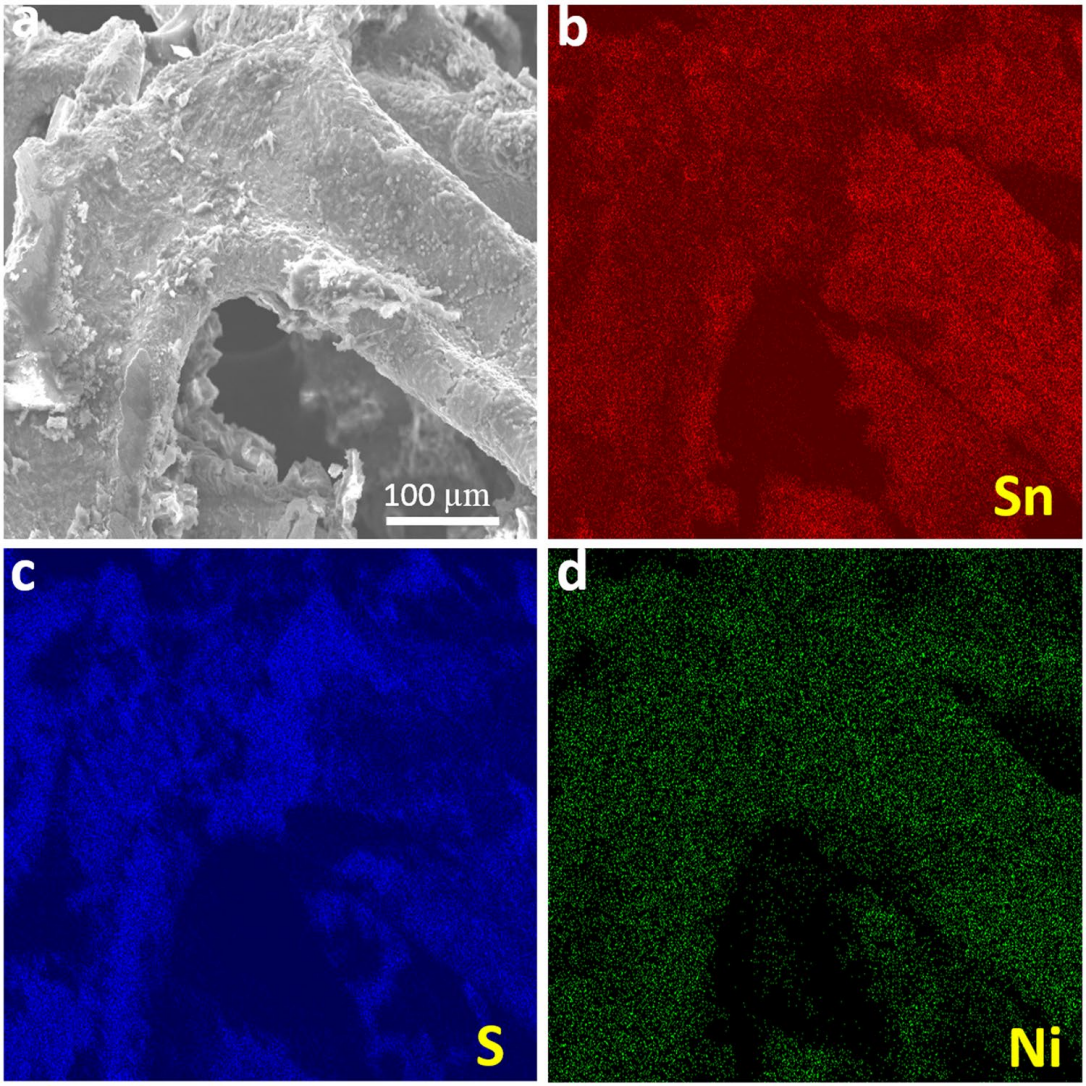
(**a**) SEM image and corresponding EDS mapping results of (**b**) Sn, (**c**) S, and (**d**) Ni for the ALD-SnS_x_@NF-160 electrode grown with 500 ALD cycles after 5000 charge/discharge cycles.

The stability of the electrode material is an essential factor to be considered in justifying the performance of energy storage devices. Generally, SnS_x_-based electrodes suffer a short cycle life, and in this context, the long-term cycling stability of the current SnS_x_@NF-160 composite electrode needs to be tested. Therefore, the cycling life test for the optimized SnS_x_@NF-160 (500 ALD cycles) was further examined by 5000 consecutive charge/discharge cycles at a fixed current density of 10 mA/cm^2^. Figure 9 displays the capacitance retention and the corresponding CE for 5000 cycles. It shows that the capacitance retention initially decreased for the first few hundred cycles, which could be attributed to the active site saturation of the surface of SnS_x_ film during the charge/discharge process. Furthermore, the capacitance retention gradually increased followed by another nominal decrease until 5000 cycles. This slight decrease in capacitance is mainly due to the structural breakdown and delamination of SnS_x_ films on the surface of NF in the aqueous solution, and the electrolyte ion may have been trapped in the layers of SnS_2_ from repeated charge/discharging^67^. Interestingly, excellent cycling stability was maintained with approximately 90% capacity retention even after 5000 cycles, and the CE was above 99% during the entire cycling process. Therefore, it was confirmed that ALD-grown SnS_x_@NF provided greater cycling stability than those of previously reported composite structure of SnS prepared by wet chemical synthesis and other methods^7,10,20,22–24^. As shown in the inset of Fig. 9, the electrochemical stability of the electrode was also evident for the 1^st^ and 5000^th^ charge/discharge processes. It is worth noting that the shape of the charge/discharge curves remained almost equivalent and nearly overlapped for the 1^st^ and 5000^th^ cycles, demonstrating superior electrochemical stability of the electrode. The capacitance retention obtained in this current work was higher than most of the earlier research reported both on SnS and other TMDC-based supercapacitors (shown in Tables S7 & S8). Such outstanding stability reflects the homogeneous and conformal deposition of a layered structure like SnS_2_ on 3D NF by ALD, with a larger interlayer spacing and suitable porosity. To elucidate the effects of consecutive charge/discharge processes on the film surface and morphology, if any, and to check the adhesion between SnS_x_ and 3D Ni-foam, a SEM analysis was performed for the samples with 5000 charge/discharge cycles. SEM-EDS mapping confirmed that after a long cycling test period, the SnS_x_ film was still uniformly presented throughout the NF, which proves the strong and robust bonding between SnS_x_ films with NF (Fig. 10). Further, the post-cycling SEM images in Fig. S12 depicted that the overall structure was still retained during the cycling process, in spite of the somewhat irregular surface agglomeration of the initial granules-like structure^13,68,69^. Therefore, outstanding cycling stability can be achieved from strong physicochemical bonding with dense layer-by-layer metal sulfide films grown by ALD on NF substrates, leading to stable mechanical and electronic contact during extensive charge/discharge processes.

## Conclusions

Unlike other TMDCs such as molybdenum disulfide (MoS_2_) and tungsten disulfide (WS_2_), tin sulfide (SnS_x_) has not been widely explored as an electrode material for supercapacitors because of its structural instability, poor conductivity issues, and low redox reactions that lead to short cycle life and lower specific capacitance. To address these issues, in this study we suggested a composite of SnS_x_@3D Ni-foam (NF) as a promising electrode for a supercapacitor. ALD processes using TDMASn and H_2_S at 180 °C and 160 °C were successfully adopted for the direct and conformal growth of SnS_x_ films on 3D NF. Electrochemical measurements systematically proved that the SnS_x_@NF-160 electrode performed better compared to the SnS_x_@NF-180. The composite electrode of SnS_x_@NF-160 demonstrated a higher areal capacitance of 805.55 mF/cm^2^ than that of SnS_x_@NF-180 (364.44 mF/cm^2^) and several other reported electrodes materials. The superior performance of SnS_x_@NF-160 is likely due to the layered structure of SnS_2_ grown at 160 °C with large inter layer spacing, which is supported by XRD, XPS, and HRTEM analyses. The precise growth per cycle, along with the self-limiting nature of ALD, permits the preparation of well-controlled SnS_x_@NF electrodes for the supercapacitor with optimum coating thicknesses, simply by varying ALD cycles. Among four electrodes grown with different ALD cycles (150, 300, 500, and 700 cycles), the SnS_x_@NF-160 electrode grown with 500 ALD cycles (with a thickness of approximately 60 nm) performed the best. An ultra-stable cycle stability up to 5000 cycles with high capacity retention (>90%) and excellent coulombic efficiency (approximately 99%) proved that this composite can be a suitable candidate for such applications. This study reveals the future promise of the ALD technique for the growth of other TDMCs to maximize the potential of composites for energy storage devices.

## Methods

### Materials synthesis

ALD-SnS_x_ films were grown on a thermally-grown silicon dioxide (SiO_2_) covered silicon (Si) wafer and 3D NF, which were used for material characterization and the supercapacitor’s electrodes, respectively, in a travelling-wave type thermal ALD reactor (Lucida D-100, NCD technology, Korea) at 0.35 Torr. For SnS_x_ film deposition, a commercially available tetrakis(dimethylamino)tin [TDMASn, ((CH_3_)_2_N)_4_Sn] and hydrogen sulfide (H_2_S) were used as an Sn precursor and a co-reactant, respectively. The TDMASn container was heated at 40 °C to provide enough vapour pressure during the SnS_x_ film deposition. High purity argon (Ar) gas (99.999%) was supplied at a flow rate of 100 sccm as a carrier gas, which facilitated the appropriate transfer of the precursor to the chamber. The following experimental protocol was applied for the SnS_x_ films deposition to guarantee the self-limiting film growth from the previous study^35^: 1 s pulsing of the TDMASn precursor, 10 s of Ar purging, 1 s pulsing of the H_2_S reactant gas, and 10 s of Ar gas purging. One ALD cycle consisted of four steps and by repeating ALD cycles, the film with a desirable thickness could be prepared precisely. The SnS_x_ films were deposited at two different process temperatures, 180 °C and 160 °C, in order to deposit SnS and SnS_2_ predominant films, respectively. NF (purity > 99.99%, with excellent anti-corrosive) is commercially available at MTI Korea and possesses distinct features, for example, light weight, highly uniform, suitable porosity (more than 95%, ~100 pores per inch. average hole diameter about 0.25 mm), intrinsic strength (Lengthwise, ≥1.25 N/mm^2^; Widthwise ≥1.00 N/mm^2^), and high thermal, electrical, and magnetic conductivities. The SnS_x_ films deposited at 180 °C and 160 °C on the NF, are henceforth abbreviated as SnS_x_@NF-180 and SnS_x_@NF-160 in this article unless stated otherwise. Further optimization of the SnS_x_@NF-160 electrode was performed by varying the SnS_x_ film’s thickness with four different (150, 300, 500, and 700) ALD cycles.

### Materials characterizations

The selected samples were examined with a plan-view scanning electron microscope (SEM, HITACHI S-4800) to characterize the morphology of the film and confirm the conformal growth of SnS_x_ on 3D NF. A cross-sectional view transmission electron microscopy (TEM, Hitachi, HF-3300 equipped with a 300 kV accelerating voltage and field emission gun) analysis was performed to study the microstructure of the film and the conformal and uniform deposition of SnS_x_ films on 3D NF. The Focused Ion Beam (FIB, Hitachi/NB 5000) technique was used to fabricate the sample for TEM analysis. An X-ray photoelectron spectroscopy (XPS, ESCALAB 250 XPS spectrometer with an Al Kα source in KBSI) analysis was performed to identify the oxidation state, chemical composition, and constituent elements of as-grown SnS_x_ films. Energy-dispersive spectroscopy (EDS), in conjunction with SEM, was used to ensure uniform elemental distribution of Sn and S on the complex 3D NF substrate.

### Electrochemical measurements

The electrochemical studies were conducted in a conventional three-electrode system, where the ALD-grown SnS_x_ films on NF (1 cm × 1 cm) directly served as working electrodes without any additional treatment. Platinum (Pt) and silver/silver chloride (Ag/AgCl) electrodes were used as the counter and reference electrodes, respectively. The electrochemical measurement of the prepared electrodes was carried out using cyclic voltammetry (CV), galvanostatic charge/discharge investigations, and electrochemical impedance spectroscopy (EIS) in a potentiostat/galvanostat (Versa STAT 3, Princeton Research, USA electrochemical workstation) instrument with aqueous potassium hydroxide (KOH) solution (2M) as the electrolyte. The areal capacitances of the prepared electrodes were calculated from the discharge characteristics region of the charge-discharge profiles using the following expression^7,13^

$${{\rm{C}}}_{{\rm{A}}}=I{t}_{{\rm{d}}}/\mathrm{VA}$$

where *I* is the current density, *t*_*d*_ is the discharge time, *V* is the potentials window, and *A* is the area of the electrode.

## Supplementary information

Supporting Information

## References

[1] Winter M, Brodd RJ (2004). What are batteries, fuel cells, and supercapacitors?. Chem. Rev..

[2] Wang G, Zhang L, Zhang J (2012). A review of electrode materials for electrochemical supercapacitors. Chem. Soc. Rev..

[3] Mahmood N, Zhang C, Yin H, Hou Y (2014). Graphene-based nanocomposites for energy storage and conversion in lithium batteries, supercapacitors and fuel cells. J. Mater. Chem. A.

[4] Simon P, Gogotsi Y, Dunn B (2014). Where do Batteries end and supercapacitors begin?. Science.

[5] Conway BE, Birss V, Wojtowicz J (1997). The role and utilization of pseudocapacitance for energy storage by supercapacitors. J. Power Sources.

[6] Borenstein A (2017). Carbon-based composite materials for supercapacitor electrodes: A review. J. Mater. Chem. A.

[7] Parveen N (2018). Facile synthesis of SnS_2_ nanostructures with different morphologies for high-performance supercapacitor applications. ACS Omega.

[8] Frackowiak E, Beguin F (2001). Carbon materials for the electrochemical storage of energy in capacitors. Carbon.

[9] Lokhande CD, Dubal DP, Joo OS (2011). Metal oxide thin film based supercapacitors. Curr. Appl. Phys..

[10] Patil AM, Lokhande AC, Shinde PA, Shelke HD, Lokhande. CD (2017). Electrochemical supercapacitor properties of SnS thin films deposited by low-cost chemical bath deposition route. I.J.E.R.T..

[11] Seo W (2017). Thickness-dependent structure and properties of SnS_2_ thin films prepared by atomic layer deposition. Jpn. J. Appl. Phys..

[12] Choi H (2018). Fabrication of high crystalline SnS and SnS_2_ thin films, and their switching device characteristics. Nanotechnology.

[13] Nandi DK (2017). Highly uniform atomic layer-deposited MoS_2_@3D-Ni-foam: A novel approach to prepare an electrode for supercapacitors. ACS Appl. Mater. Interfaces.

[14] Ansari MZ, Ansari SA, Parveen N, Cho MH, Song T (2018). Lithium ion storage ability, supercapacitor electrode performance, and photocatalytic performance of tungsten disulfide nanosheets. New J. Chem..

[15] Ahn JH (2015). Deterministic two-dimensional polymorphism growth of hexagonal n-type SnS_2_ and orthorhombic p-type SnS crystals. Nano Lett..

[16] Piacente V, Foglia S, Scardala P (1991). Sublimation study of the tin sulphides SnS_2_, Sn_2_S_3_ and SnS. J. Alloys Compd..

[17] Chia X, Lazar P, Sofer Z, Luxa J, Pumera M (2016). Layered SnS versus SnS_2_: valence and structural implications on electrochemistry and clean energy electrocatalysis. J. Phys. Chem.C.

[18] Sun W (2015). Two-dimensional tin disulfide nanosheets for enhanced sodium storage. ACS Nano.

[19] Wu. P (2012). Layer- stacked tin disulfide nanorods in silica nanoreactors with improved lithium storage capabilities. Nanoscale.

[20] Chauhan H, Singh MK, Hashmi SA, Deka S (2015). Synthesis of surfactant-free SnS nanorods by a solvothermal route with better electrochemical properties towards supercapacitor applications. RSC Adv..

[21] Ravuri S, Pandey CA, Ramchandran R, Jeon SK, Grace AN (2017). Wet chemical synthesis of SnS/graphene nanocomposites for high performance supercapacitor electrodes. Int. J. Nanosci..

[22] Wang L, Ma Y, Yang M, Qi Y (2015). One-pot synthesis of 3D flower-like heterostructured SnS_2_/MoS_2_ for enhanced supercapacitor behavior. RSC Adv..

[23] Mishra RK, Baek GW, Kim K, Kwon H, Jin SH (2017). One-step solvothermal synthesis of carnation flower-like SnS_2_ as superior electrodes for supercapacitor applications. Appl. Surf. Sci..

[24] Chauhan H, Singh MK, Kumar P, Hashmi SA, Deka S (2016). Development of SnS_2_/RGO nanosheet composite for cost-effective aqueous hybrid supercapacitors. Nanotechnology.

[25] Li Y, Xie H, Wang J (2011). Preparation and electrochemical performances of carbon-coated nanoscale SnS for supercapacitors. J.Solid State Electr..

[26] Ma L, Xu L, Zhou X, Xu X, Zhang L (2015). Molybdenum-doped few-layered SnS_2_ architectures with enhanced electrochemical supercapacitive performance. RSC Adv..

[27] Turan E, Kul M, Aybek AS, Zor M (2009). Structural and optical properties of SnS semiconductor films produced by chemical bath deposition. J. Phys. D: Appl. Phys..

[28] Hartman K (2011). SnS thin-films by RF sputtering at room temperature. Thin Solid Films.

[29] Miles RW, Ogah OE, Zoppi G, Forbes I (2009). Thermally evaporated thin films of SnS for application in solar cell devices. Thin Solid Films.

[30] Ichimura M, Takeuchi K, Ono Y, Arai E (2000). Electrochemical deposition of SnS thin films. Thin Solid Films.

[31] Lopez S, Ortiz A (1994). Spray pyrolysis deposition of Sn_x_S_y_ thin films. Semicond. Sci. Technol..

[32] Liu SA (2010). Chemical bath deposition of SnS_2_ nanowall arrays with improved electrochemical performance for lithium ion battery. Mater. Lett..

[33] Manasevit HM, Simpson WI (1975). The use of metalorganics in the preparation of semiconductor materials VI. formation of IV–VI lead and tin salts. J. Electrochem. Soc..

[34] Sinsermsuksakul P, Heo J, Noh WA, Hock S, Gordon RG (2011). Atomic layer deposition of tin monosulfide thin films. Adv. Energy Mater..

[35] Jang B, Yeo S, Kim H, Shin B, Kim SH (2017). Fabrication of single-phase SnS film by H_2_ annealing of amorphous SnS_x_ prepared by atomic layer deposition. J. Vac. Sci. Technol. A.

[36] Sinsermsuksakul P (2014). Overcoming efficiency limitations of SnS-based solar cells. Adv. Energy Mater..

[37] George SM (2010). Atomic layer deposition: an overview. Chem. Rev..

[38] Liu Y (2015). SnO_2_ coated carbon cloth with surface modification as Na-ion battery anode. Nano Energy.

[39] Bilousov OV (2017). Atomic layer deposition of cubic and orthorhombic phase tin Monosulfide. Chem. Mater..

[40] Ham G (2013). Tuning the electronic structure of tin sulfides grown by atomic layer deposition. ACS Appl. Mater. Interfaces.

[41] Baek IH (2017). Synthesis of SnS thin films by atomic layer deposition at low temperatures. Chem. Mater..

[42] Boukhalfa S, Evanoff K, Yushin G (2012). Atomic layer deposition of vanadium oxide on carbon nanotubes for high-power supercapacitor electrodes. Energ Environ. Sci..

[43] Kao E, Yang C, Warren R, Kozinda A, Lin L (2016). ALD titanium nitride on vertically aligned carbon nanotube forests for electrochemical supercapacitors. Sens Actuators A Phys..

[44] Sun X (2012). Atomic layer deposition of TiO_2_ on graphene for supercapacitors. J. Electrochem. Soc..

[45] Yu L (2016). Highly effective synthesis of NiO/CNT nanohybrids by atomic layer deposition for high-rate and long-life supercapacitors. Dalton Trans..

[46] Warren R, Sammoura F, Tounsi T, Sanghadasa M, Lin L (2015). Highly active ruthenium oxide coating via ALD and electrochemical activation in supercapacitor applications. J. Mater. Chem. A.

[47] Li H, Gao Y, Shao Y, Su Y, Wang X (2015). Vapor-phase atomic layer deposition of Co_9_S_8_ and its application for supercapacitors. Nano Lett..

[48] Wen L (2014). Cost-effective Atomic Layer Deposition Synthesis of Pt Nanotube Arrays: Application for High Performance Supercapacitor. Small.

[49] Chen C (2014). NiO/nanoporous graphene composites with excellent supercapacitive performance produced by atomic layer deposition. Nanotechnology.

[50] Wanga R, Xia C, Wei N, Alshareef HN (2016). NiCo_2_O_4_@TiN Core-shell electrodes through conformal atomic layer deposition for all-solid-state supercapacitors. Electrochim. Acta.

[51] Chandrasekhar HR, Humphreys RG, Zwick U, Cardona M (1977). Infrared and Raman spectra of the IV-VI compounds SnS and SnSe. Phys. Rev. B.

[52] Devika M (2010). The physical properties of SnS films grown on lattice-matched and amorphous substrates. Phys. Status Solidi A.

[53] Reddy NK (2013). Growth-temperature dependent physical properties of SnS nanocrystalline thin films. ECS J. Solid State Sci. Technol..

[54] Mathews NR, Anaya HBM, Jacome MAC, Chavez CA, Antoniob JAT (2010). Tin sulfide thin films by pulse electrodeposition: structural, morphological, and optical properties. J. Electrochem. Soc..

[55] Kılıc C, Zunger A (2002). Origins of coexistence of conductivity and transparency in SnO_2_. Phys. Rev. Lett..

[56] Zang X (2014). Evaluation of Layer-by-layer graphene structures as supercapacitor electrode materials. J. Appl. Phys..

[57] Nandi DK (2018). Low temperature atomic layer deposited molybdenum nitride-Ni-foam composite: an electrode for efficient charge storage. Electrochem. Commun..

[58] Ratha S, Rout CS (2013). Supercapacitor electrodes based on layered tungsten disulfide-reduced graphene oxide hybrids synthesized by a facile hydrothermal method. ACS Appl. Mater. Interfaces.

[59] Balasingam SK, Lee JS, Jun Y (2015). Few-layered MoSe_2_ nanosheets as an advanced electrode material for supercapacitors. Dalton Trans..

[60] Liu C (2017). 3D porous nanoarchitectures derived from SnS/S-doped graphene Hybrid Nanosheets for Flexible All-Solid-State Supercapacitors. Small.

[61] Jayalakshmi M, Rao MM, Choudary BM (2004). Identifying nano SnS as a new electrode material for electrochemical capacitors in aqueous solutions. Electrochem. Commun..

[62] Patil AM, Lokhande VC, Patil UM, Shinde PA, Lokhande CD (2018). High performance all-solid-state asymmetric supercapacitor device based on 3D nanospheres of β-MnO_2_ and Nanoflowers of O-SnS. ACS Sustain Chem. Eng..

[63] Yan J (2012). Fabrication and electrochemical performances of hierarchical porous Ni(OH)_2_ nanoflakes anchored on graphene sheets. J.Mater. Chem..

[64] Shao J (2015). Mechanism analysis of the capacitance contributions and ultra long cycling-stability of the isomorphous MnO_2_@MnO_2_ core/shell nanostructures for supercapacitors. J. Mater. Chem. A.

[65] Du D, Lan R, Xie K, Wang H, Tao S (2017). Synthesis of Li_2_Ni_2_(MoO_4_)_3_ as a high-performance positive electrode for asymmetric supercapacitors. RSC Adv..

[66] Sankar KV (2015). Studies on the electrochemical intercalation/de-intercalation mechanism of NiMn_2_O_4_ for high stable pseudocapacitor electrodes. RSC Adv..

[67] Shinde NM, Xia QX, Yun JM, Mane RS, Kim KH (2018). Polycrystalline and mesoporous 3-D Bi_2_O_3_ nanostructured negatrodes for high-energy and power asymmetric supercapacitors: superfast room temperature direct wet chemical growth. ACS Appl. Mater. Interfaces.

[68] Dhara A, Sarkar SK, Mitra S (2017). Controlled 3D carbon nanotube architecture coated with MoO_x_ material by ALD technique: A high energy density lithium-ion battery electrode. Adv. Mater. Interfaces.

[69] Li B (2015). Hollow carbon nanospheres/silicon/alumina core-shell film as an anode for lithium-ion batteries. Sci. Rep..

