# Supplementary material for: Enhanced activity of highly conformal and layered tin sulfide (SnSx) prepared by atomic layer deposition (ALD) on 3D metal scaffold towards high performance supercapacitor electrode
Source: Sci Rep. 2019 Jul 15;9:10225. doi: 10.1038/s41598-019-46679-7 (PMC6629880; doi:10.1038/s41598-019-46679-7)
Supplement: Supplementary file 1 — Supporting Information [file 41598_2019_46679_MOESM1_ESM.docx]

Supporting Information

**Enhanced activity of highly conformal and layered tin sulfide (SnSx) prepared by atomic layer deposition (ALD) on 3D metal scaffold towards high performance supercapacitor electrode**

Mohd Zahid Ansari ,^[a]^ Nazish Parveen, ^[b]^ Dip K. Nandi,^[a]^ Rahul Ramesh,^[a^

^]^ Sajid Ali Ansari, ^[c]^ Taehoon Cheon,^[a,d]^,and Soo-Hyun Kim ^[a], *^

[a] School of Materials Science and Engineering, Yeungnam University, Gyeongsan 712-749, Republic of Korea

[b] Department of Chemistry, College of Science, King Faisal University, Kingdom of Saudi Arabia

[c] Department of Physics, College of Science, King Faisal University, Kingdom of Saudi Arabia

[d] Center for Core Research Facilities, Daegu Gyeongbuk Institute of Science & Technology, Sang-ri, Hyeonpung-myeon, Dalseong-gun, Daegu, 711-873, Republic Korea

*Corresponding author email: [soohyun@ynu.ac.kr](mailto:soohyun@ynu.ac.kr)

**Table S1.** Comparison of ALD-based active electrode materials for supercapacitor.

| **Sr.no.** | | **Electrode materials** | **Current density** | **Areal capacitance/specific capacitance** | **Cycle no.** | **Retention**  **(%)** | **References** |
| --- | --- | --- | --- | --- | --- | --- | --- |
| **1**  **2**  **3**  **4**  **5**  **6**  **7**  **8**  **9**  **10**  **11**  **12** | Vo_x_@CNT  TiN-CNT  TiO_2_-G  NiO/CNT  RuO_x_-CNT  Co_9_S_8_/NF  Pt-NT  NiO/NG  NiCo_2_O_4_@TiN  MoS_2_@3D-Ni-foam  MoN_x_@NF  SnS_x_@NF-160 | | 1A/g  -  10mV/s  2A/g  -  3A/g  5mV/s  1A/g  1 mA/cm^2^  3mA/cm^2^  2 mA/cm^2^  0.5 mA/cm^2^ | 1550F/g  81mF/cm^2^  84F/g  2013 F/g  644F/g  1645F/g  810 Fg-1 and 75 mF cm^-2^  1897.1F/g  82mF/cm^2^  3400mF/cm^2^  130mC/cm^2^  805.55mF/cm^2^ | 5000 at 5A/g  300  1000 at 2A/g  4000 at10A/g  10000 at 100 mV/s  2000 at 45A/g  8000 at 2 to 100A/g  1500 at 2A/g  20000 at 10 mA/cm^2^    4500 at 50mA/cm^2^  8000  5000 at 10 mA/cm^2^ | 92  90  87.5  74  -  -  68  94  70  80  115  90 | 42  43  44  45  46  47  48  49  50  13  68  Present study |

**Table S2.** Structural parameters determined by X-ray diffraction analysis.


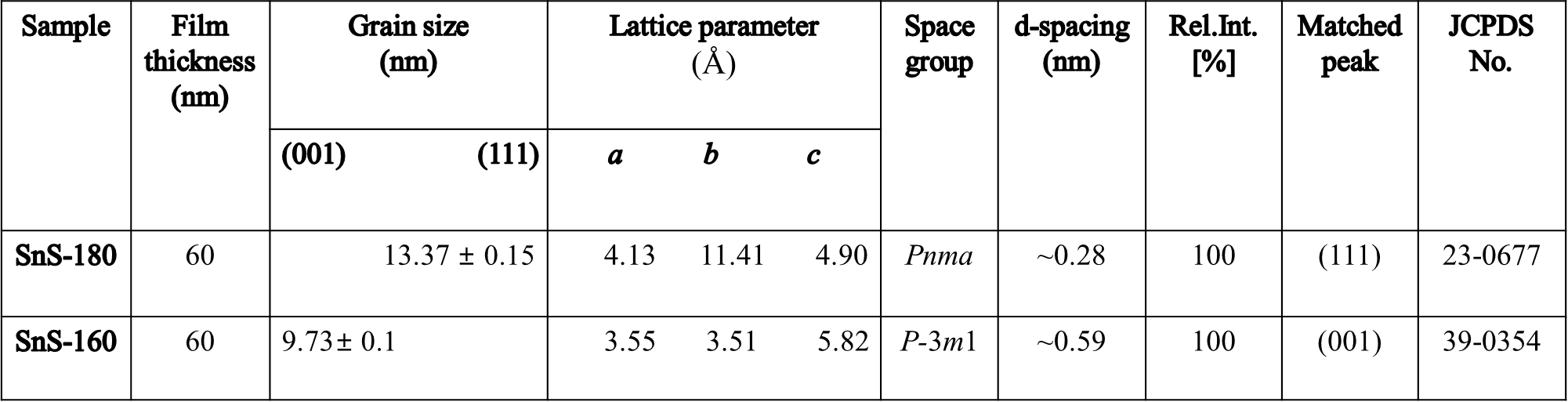


**
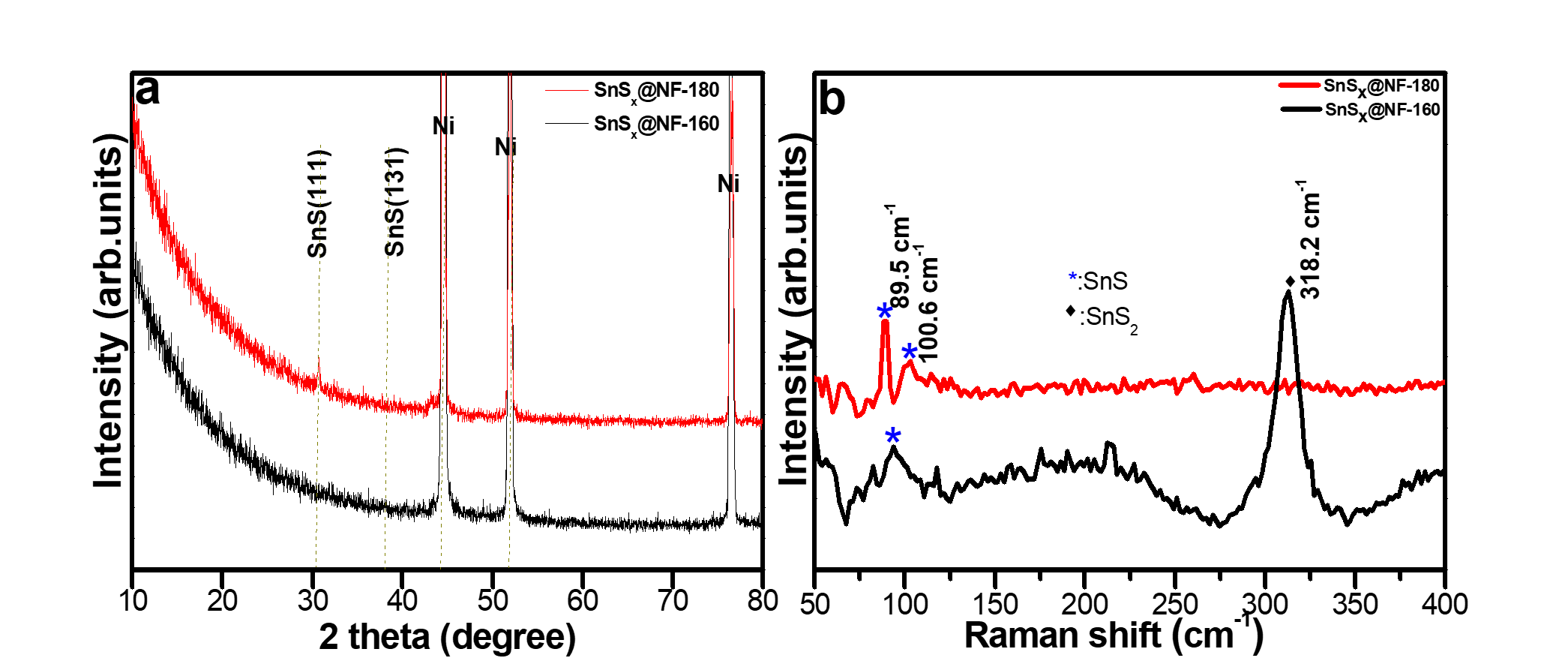
**

**Figure S1.** (a) XRD patterns and (b) Raman spectroscopy of the as-grown ALD-SnS_x_ thin films grown on the NF by 500 cycles.

**
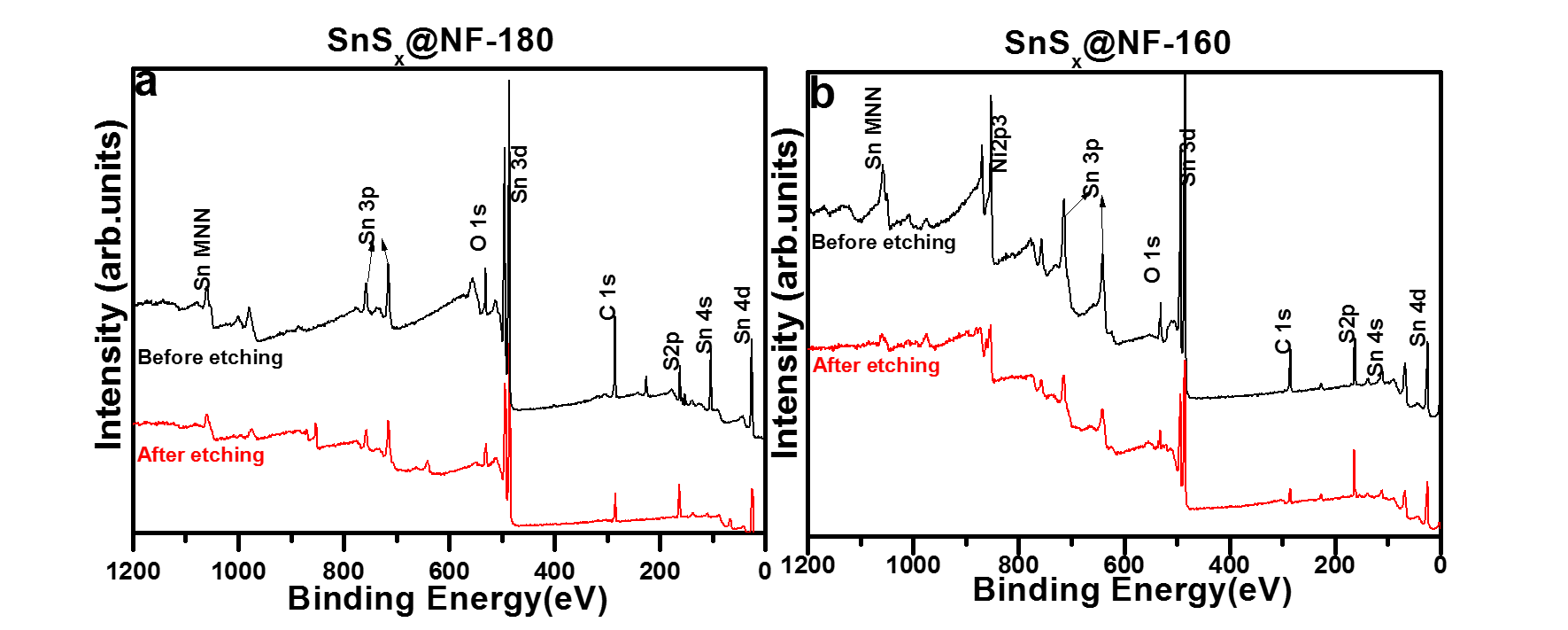
**

**Figure S2.** Full XPS survey spectra of SnS_x_ film before and after Ar-ion etching (a) SnS_x_@NF-180 and (b) SnS_x_@NF-160.

**Table S3.** Atomic concentrations of SnS_x_@NF-180 and SnS_x_@NF-160 from full XPS spectra.

**
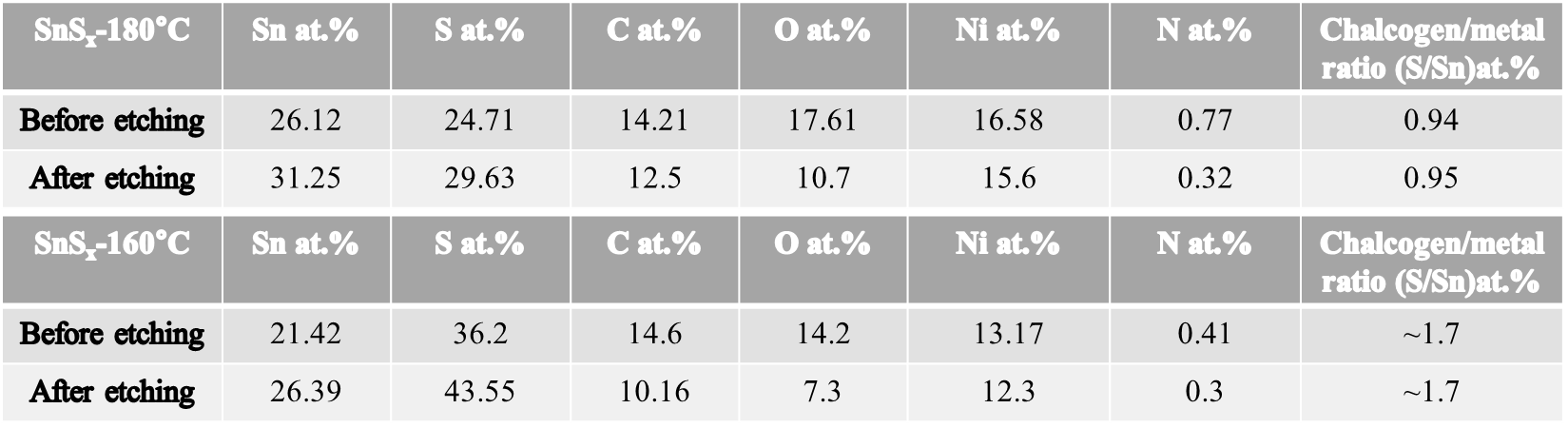
**

**
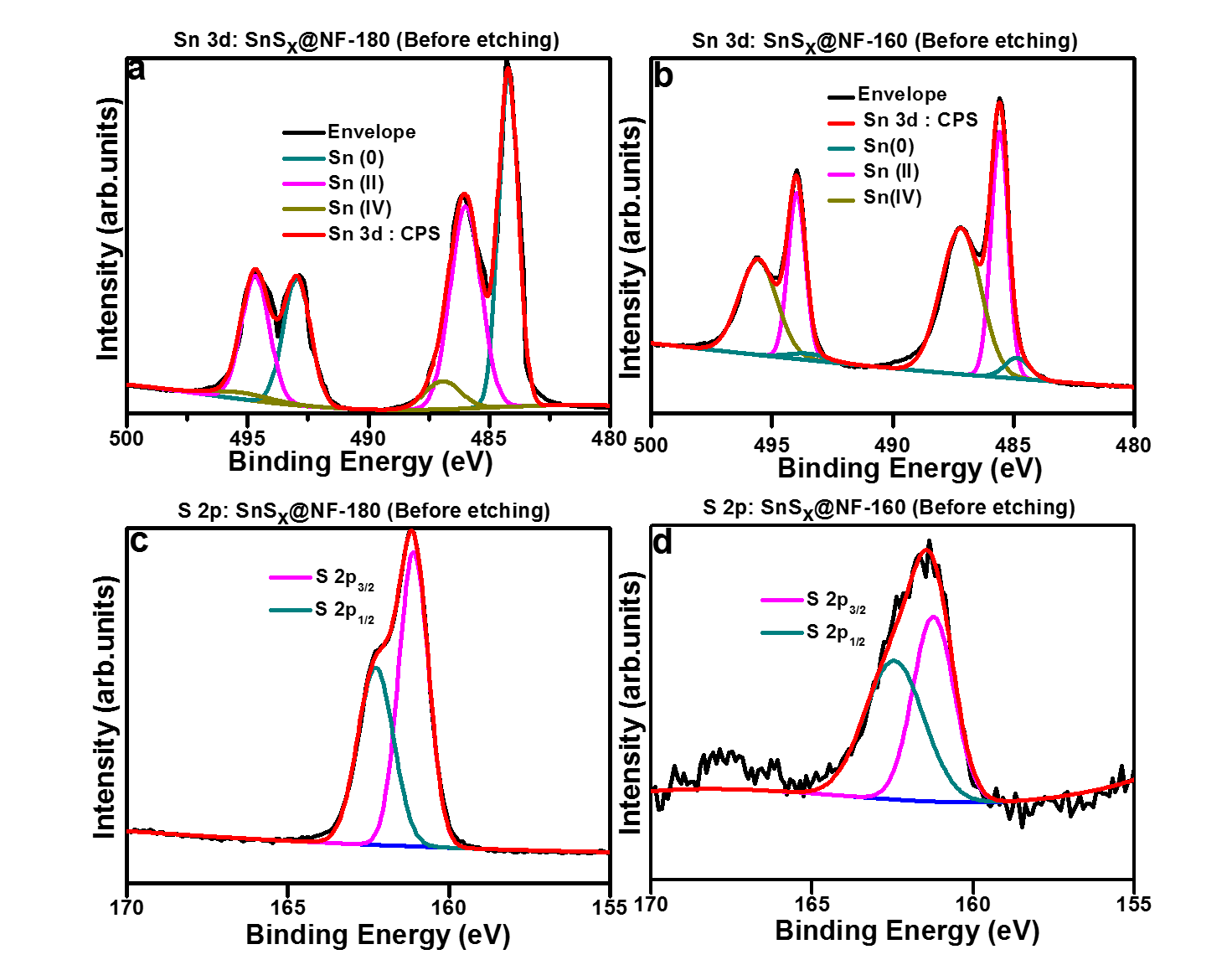
**

**Figure S3.** X-ray photoelectron spectroscopy spectra of Sn 3d region for (a) SnS_x_@NF-180, and (b) SnS_x_@NF-160, S 2p region for (c) SnS_x_@NF-180, and (d) SnS_x_@NF-160.

**Table S4.** Table of weight percentages of Sn with 0, +2 and +4 valencies in SnS_x_-@NF-180 and SnS_x_-@NF-160 determined from high-resolution XPS.

**
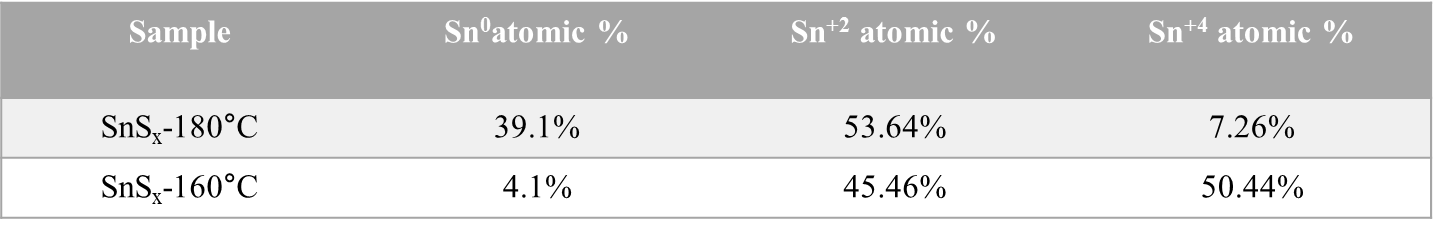
**


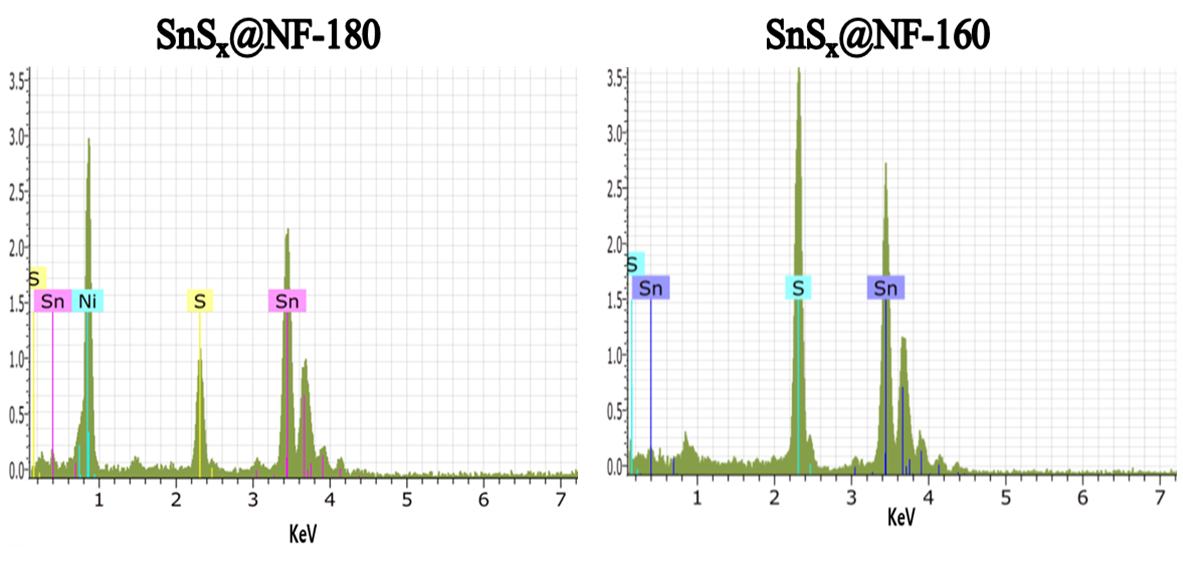
**Figure S4.** Energy dispersive X-ray spectra of SnS_x_@NF-180 and SnS_x_@NF-160.

**Table S5.** Atomic concentration of Sn chalcogenides based on energy-dispersive X-ray spectroscopy. Chalcogen-to-metal ratios are also computed for SnS_x_@NF-180 and SnS_x_@NF-160.

**
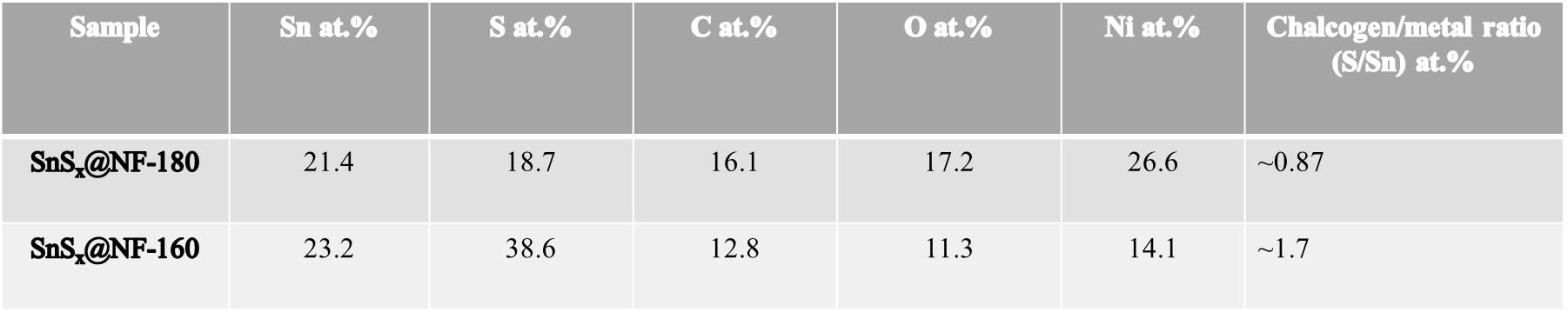
**


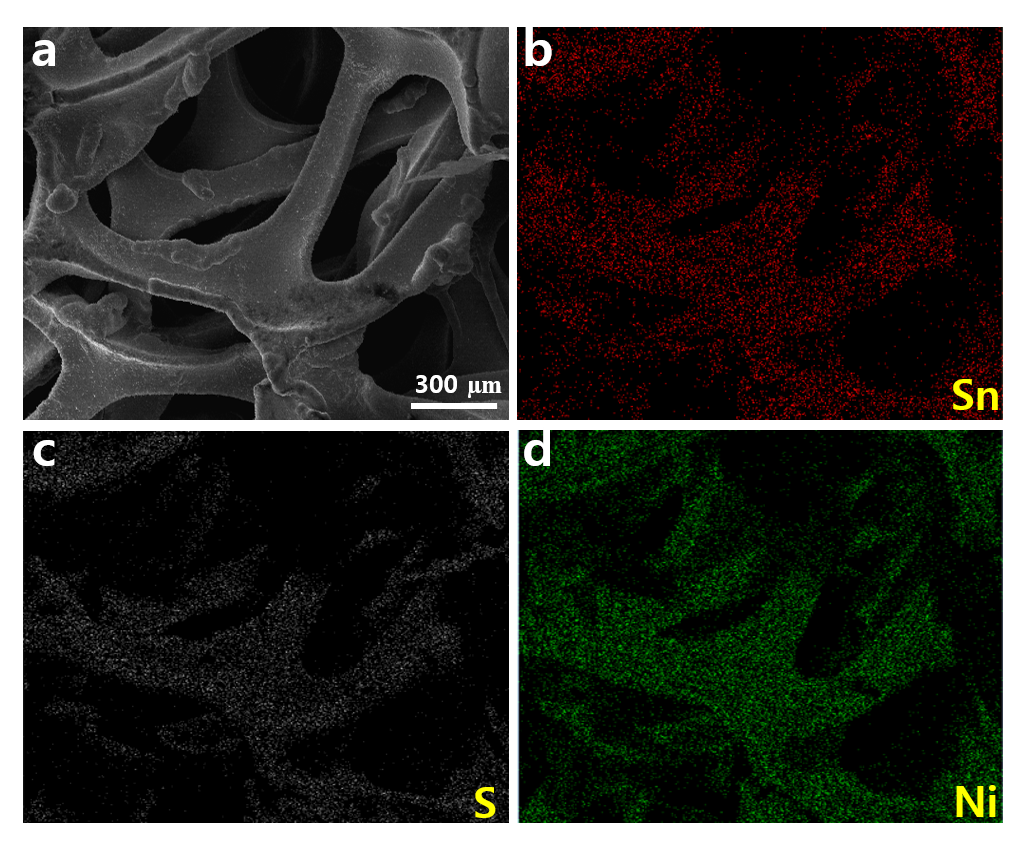


**Figure S5.** (a) SEM image of the ALD-SnS_x_@NF-160, (b) corresponing EDS elemetal mapping for Sn, (c) S, and (d) Ni showing the uniform distribution on the NF.


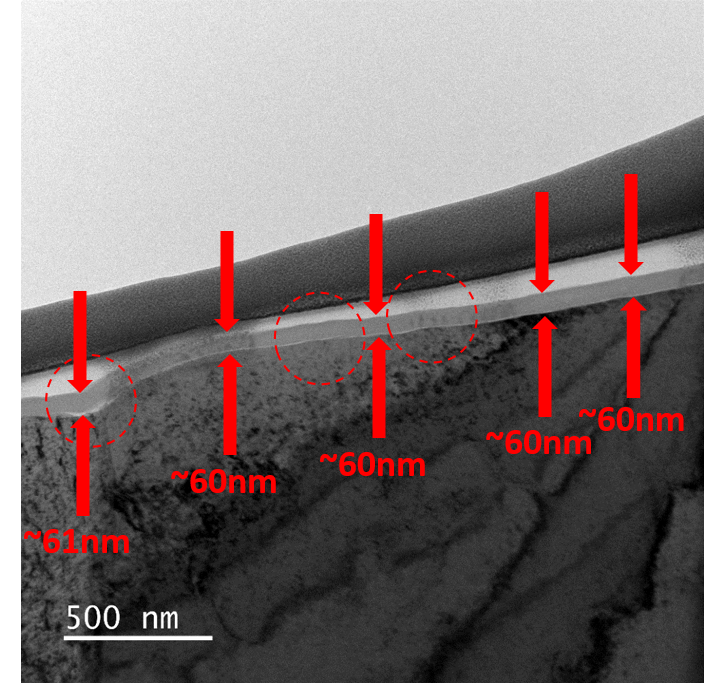


**Figure S6 .** Cross-sectional view TEM images of the SnS_x_@NF-160 electrode grown by 500 ALD cycles to show the uniform thicknesses measured at the different regions.


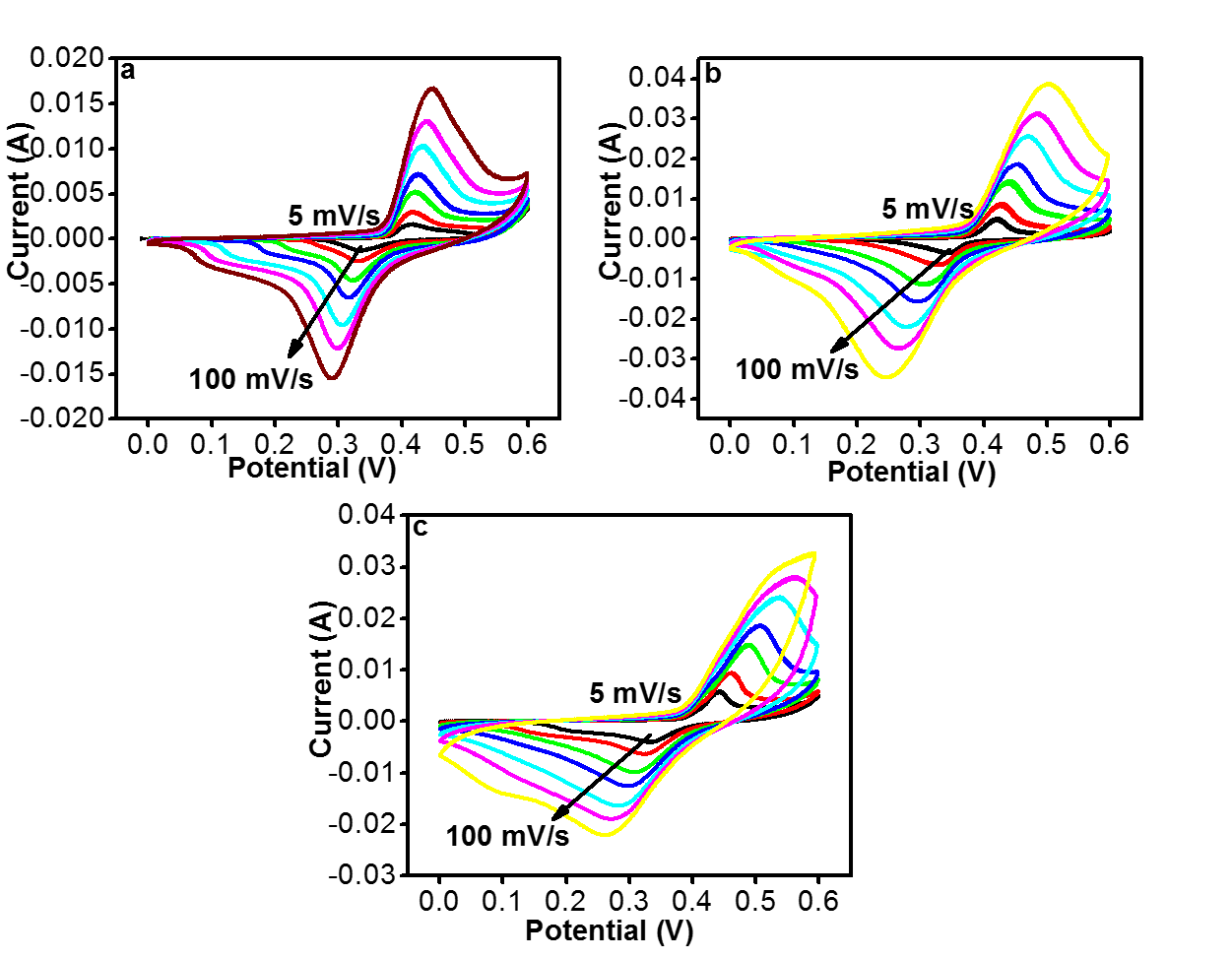


**Figure S7.** CV curves at different scan rates for the ALD-SnS_x_@NF-160 electrode grown by (a) 150, (b) 300, and (c) 700 ALD cycles.


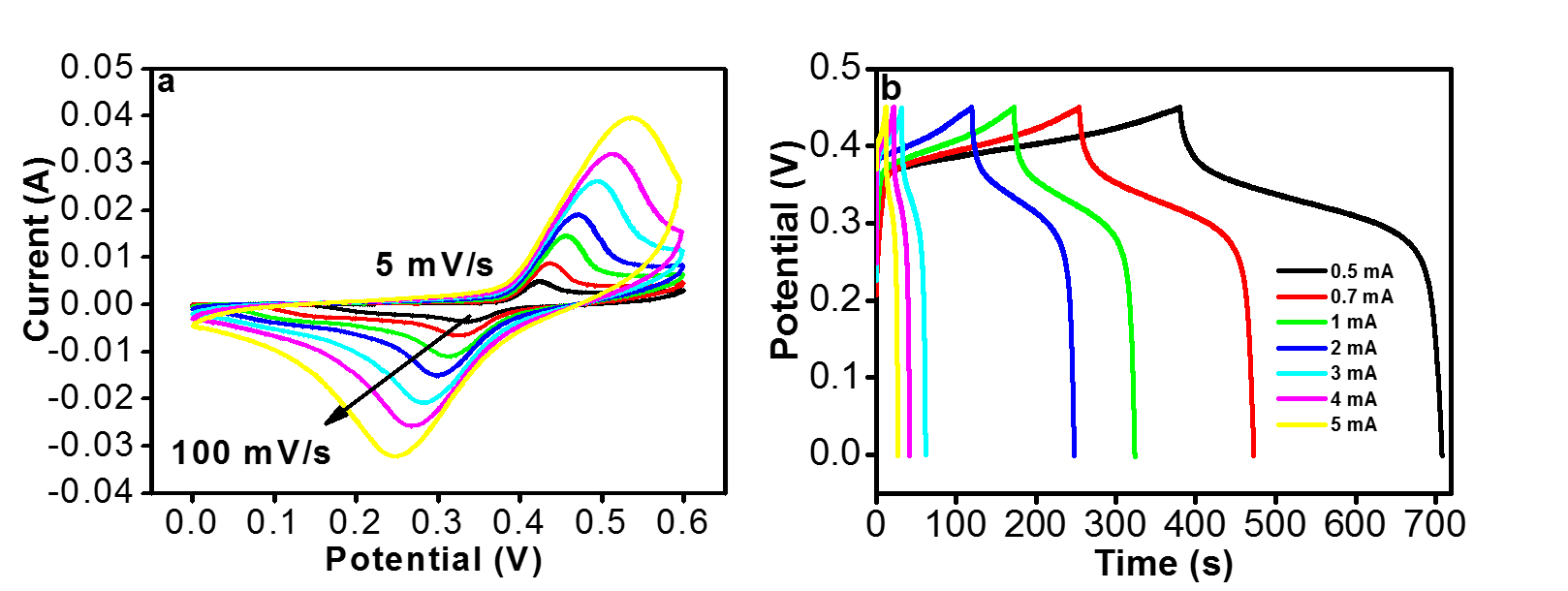


**Figure S8.** (a) CV curves and (b) charge-discharge profile at different scan rates for the ALD-SnS_x_@NF-180 electrode.


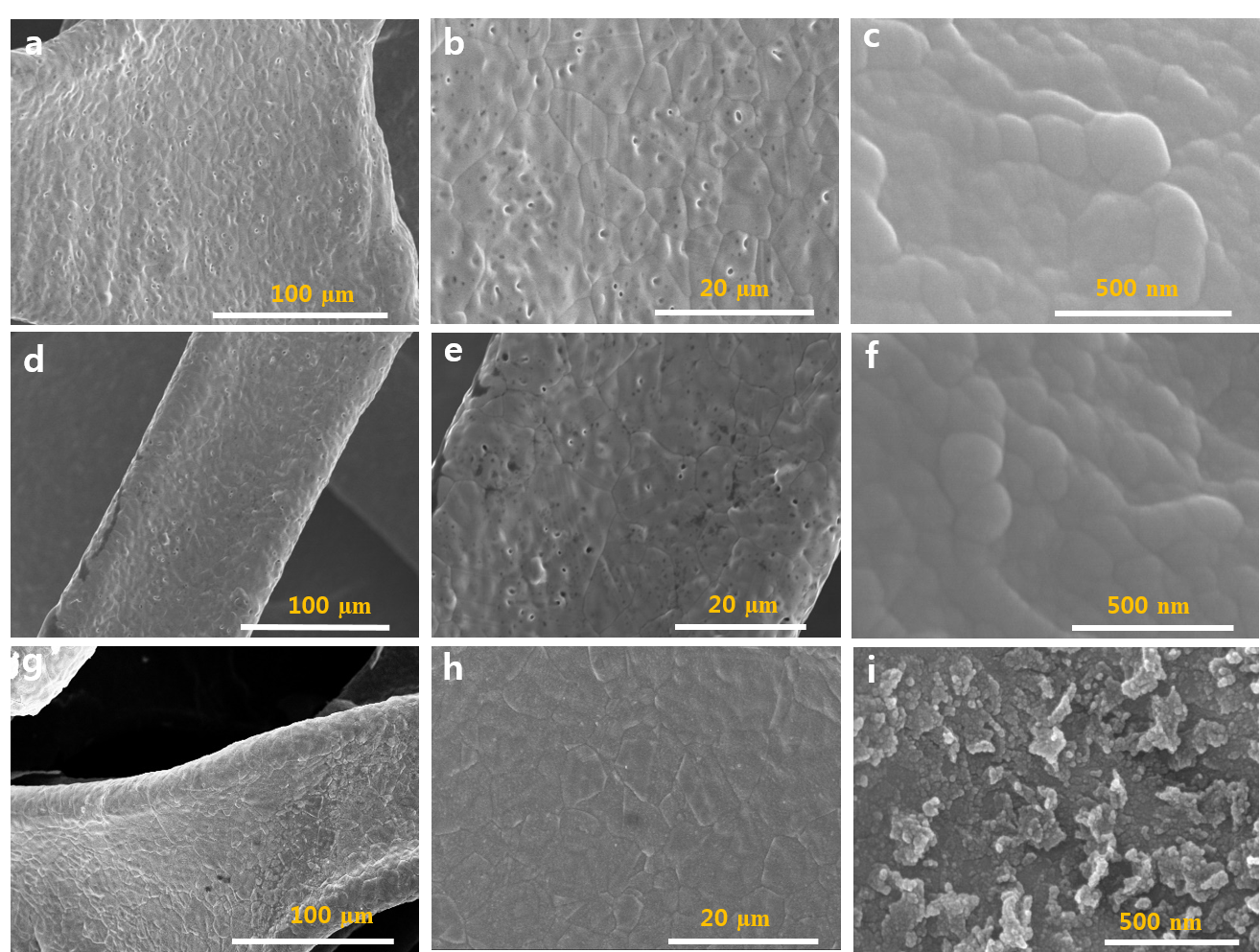


**Figure S9.** SEM images of the SnS_x_@NF-160 electrode prepared with different ALD cycles of (a-c) 150, (d-f) 300, and (g-i) 700 ALD cycles.


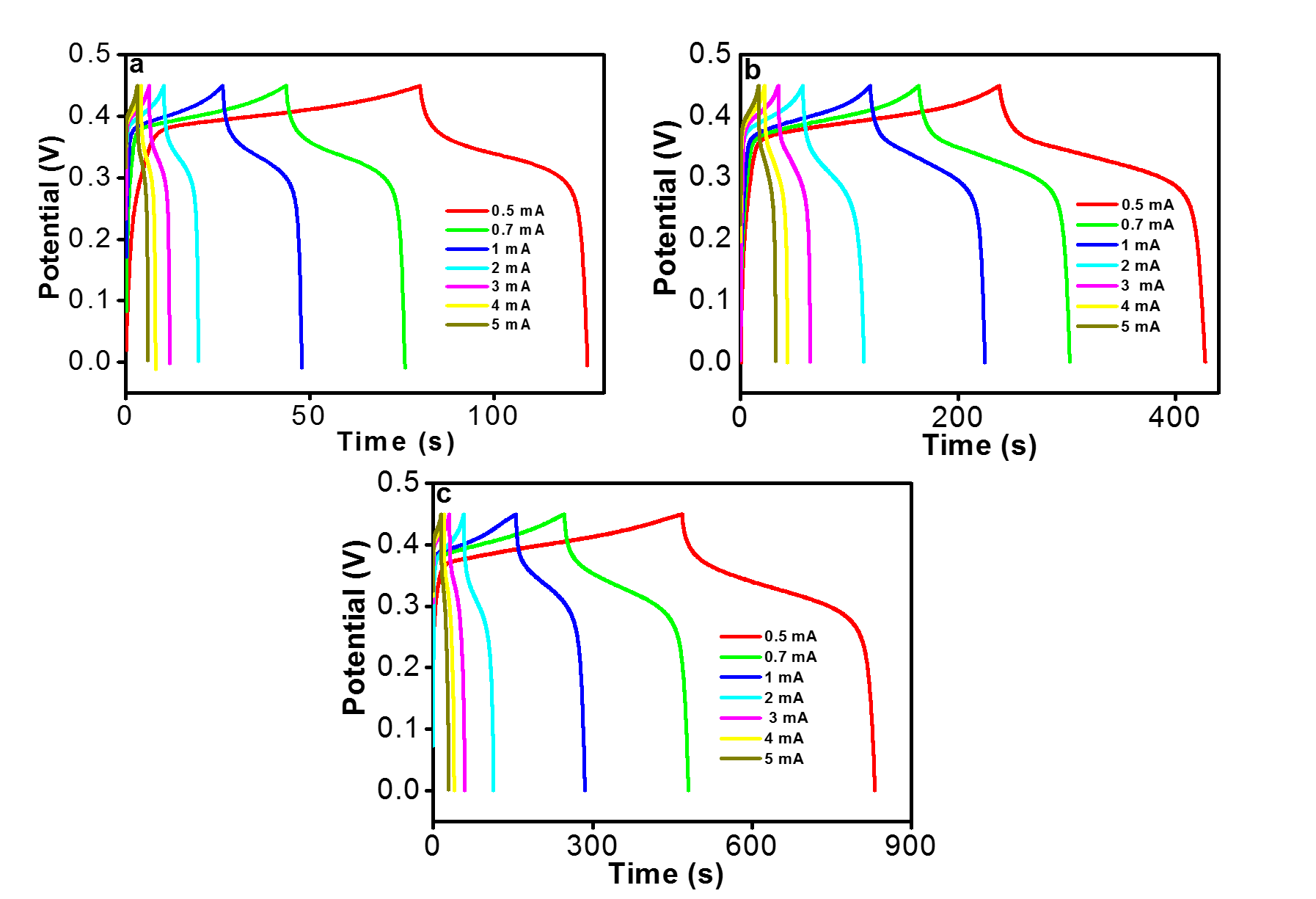


**Figure S10.** Charge-discharge profiles at different current densities for ALD-SnS_x_@NF-160 electrode grown by (a) 150, (b) 300, and (c) 700 ALD cycles.


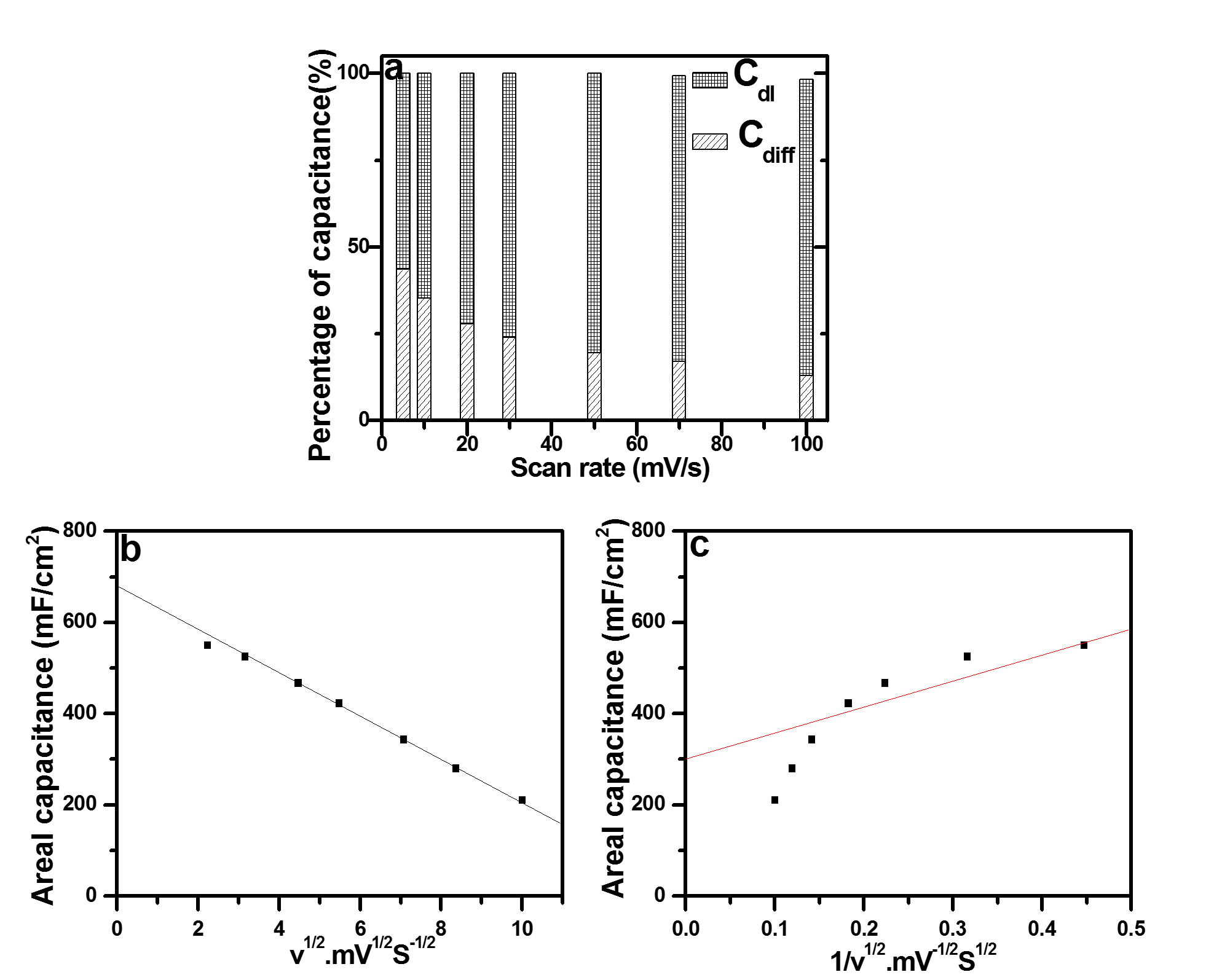


**Figure S11**. (a) areal capacitance contribution in percentage, Trasatti plots: (b) areal capacitance vs. square root of scan rate, and (c) areal capacitance vs. reciprocal of the square root of scan rate.


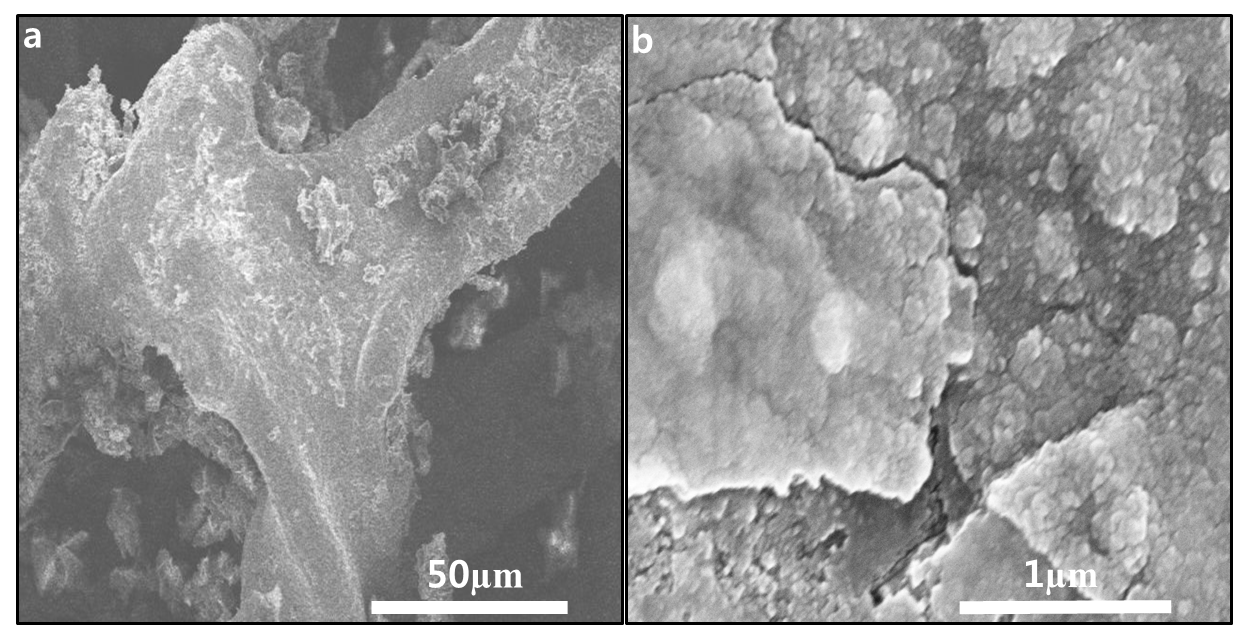


**Figure S12.** SEM images of the ALD-SnS_x_-160@Ni-foam electrode grown by 500 ALD cycles after 5000 charge-discharge cycles at (a) low and (b) high magnification of a selected portion.

**Table S6.** Comparison of areal capacitance of electrode materials for supercapacitor.

| **Sr.no.** | | **Electrode materials** | **Preparation method** | **Arial capacitance** | **Current density** | **Cycle no.** | **Retention**  **(%)** | | **Refs.** |
| --- | --- | --- | --- | --- | --- | --- | --- | --- | --- |
| **1**  **2**  **3**  **4**  **5**  **6**  **7**  **8**  **9**  **10**  **11**  **12**  **13** | MSC  MoS_2_-Graphene  NiO/MnO_2_  1*H-*MoS_2_@oleylamine  MoS_2_/CoS_2_  MoS_2_@CNT/RGO  graphene oxide/MnSe_2_  1T-MoS_2_  MoS_2_/PPy  MoS_2_ film  MoS_2_ thin film  NiO/MnO2@carbon cloth  SnS_x_@NF-160 | | Laser pattering  exfoliation  Hydrothermal  hot-injection thermolysis  Hydrothermal  Hydrothermal  Hydrothermal  Exfoliation  Oxidative polymerization  spray painting  CVD  Hydrothermal  ALD | 15.6 mF/cm^2^ at 10mV/s  11 mF/cm^2^  316.3 mF/cm^2^  50.6 mF/cm^2^  142.5 mF/cm^2^  129 mF/cm^2^  93.3 mF/cm^2^  1.1 mF/cm^2^  0.31 mF/cm^2^  8 mF/cm^2^  71 mF/cm^2^  286 mF/cm^2^  805.55 mF/cm^2^ | -  1mA/cm^2^  10 mA/cm^2^  2.75 mA/cm^2^  1 mA/cm^2^  10 mA/cm^2^  0.3 mA/cm^2^  0.04 mA/cm^2^  1 A/g  0.02 mA/cm^2^  1mV/s  0.5 mA/cm^2^  0.5mA/cm^2^ | 2000  10000  2200  5000  1000  10000  4500  5000  4000  1000  -  2200  5000 | 83  -  89  250  92.7  94.7  80  130.6  85  92  -  89  90 | S13  S14  S15  S16  S7  S17  S18  S19  S20  S21  S22  S15  this  work | |

**Table S7.** Comparison of cycling stabilities of different SnS electrodes.

| **Sr.no.** | | **Electrode materials** | **Preparation method** | **Cycle no.** | **Retention**  **(%)** | **References** |
| --- | --- | --- | --- | --- | --- | --- |
| **1**  **2**  **3**  **4**  **5**  **6**  **7**  **8**  **9**  **10**  **11**  **12**  **13**  **14** | SnS-CB composite  Nano SnS  m-SnS_2_  SnS_2_/RGO  CF-SnS_2_  SnS_2_/MoS_2_  SnS nanorods  SnS  SnS_2_ nanosheets  SnS_2_ nanoparticles  SL-SnS_2_  EL-SnS_2_  FL-SnS_2_  SnS_x_@NF-160 | | Hydrothermal  Hydrothermal  Hydrothermal  Wet chemical process  Hydrothermal  Hydrothermal  Hydrothermal  CBD  Hydrothermal  Wet chemical process  Hydrothermal  Hydrothermal  Hydrothermal  ALD | 1000  1000  1000  1000  1000  1000  500  2000  1000  500  2000  2000  2000  5000 | 80  -  89  95  68  94.4  60  89  -  95  80  82  90  90 | 50  48  26  24  23  22  20  10  26  24  07  07  07  Present study |

**Table S8.** Comparison of cyclic performances of other metal sulfide based electrode materials for supercapacitor.

| **Sr.no.** | | **Electrode materials** | **Preparation method** | **Cycle no.** | **Retention**  **(%)** | **References** |
| --- | --- | --- | --- | --- | --- | --- |
| **1**  **2**  **3**  **4**  **5**  **6**  **7**  **8**  **9**  **10**  **11**  **12**  **13** | PANI/MoS_2_  Sphere MoS_2_  Cu_2_SnS_3_  Ni-Co sulfide  CuS  CuS network  MoS_2_/CoS_2_  NiS  WS_2_/SWCNT  WS_2_/CFC  WS_2_NP/CTs  MoS_2_/C  SnS_x_@NF-160 | | Polymerization  Hydrothermal  Hydrothermal  Hydrothermal  Hydrothermal  Hydrothermal  Hydrothermal  CBD  Hydrothermal  Wet chemical process  Hydrothermal  Hydrothermal  ALD | 500  1000  2000  3000  2000  1500  1000  3000  500  500  500  1000  5000 | 98  93.8  60  78.5  90.7  80.5  92.7  85.3  45  99  42  89.6  90 | S1  S2  S3  S4  S5  S6  S7  S8  S9  S10  S11  S12  Present study |

References (S1-S22)

1. Huang, K.J., Wang, L., Liu, Y.J., Wang, H.B., Liu, Y.M. & Wang, L.L. Synthesis of polyaniline/2-dimensional graphene analog MoS_2_ composites for high-performance supercapacitor. . *Electrochim. Acta* **109**, 587-594 (2013).
2. Krishnamoorthy, K., Veerasubramani, G.K., Radhakrishnan, S. & Kim, S. J. Supercapacitive properties of hydrothermally synthesized sphere like MoS_2_ nanostructures. *Mater. Res. Bull.* **50**, 499-502 (2014).
3. Wang, C., Tian, H., Jiang, J., Zhou, T., Zeng, Q., He, X. R., Huang, P. & Yao, Y. Facile synthesis of different morphologies of Cu_2_SnS_3_ for high-performance supercapacitors. *ACS Appl. Mater. Interfaces* **9**, 26038–26044 (2017).
4. Li,Y., Cao,L., Qiao, L., Zhou, M., Yang, Y. Xiao, P. & Zhang, Y. Ni–Co sulfide nanowires on nickel foam with ultrahigh capacitance for asymmetric supercapacitors. *J. Mater. Chem. A* **2**, 6540-6548 (2014).
5. Zhang,Y., Xu,J., Zheng,Y., Hu,X., Shang,Y. & Zhang,Y. Interconnected CuS nanowalls with rough surfaces grown on nickel foam as high-performance electrodes for supercapacitors. *RSC Adv.* **6**, 59976-59983 (2016).
6. Fu, W., Han, W., Zha, H., Mei, J., Li, Y., Zhang, Z. & Xie, E. Nanostructured CuS networks composed of interconnected nanoparticles for asymmetric supercapacitors. *Phys. Chem. Chem. Phys.* **18**, 24471-24476 (2016).
7. Wang, L., Zhang, X., Ma,Y., Yang, M. & Qi, Y. Supercapacitor performances of the MoS_2_/CoS_2_ nanotube arrays in situ grown on Ti plate. *J. Phys. Chem. C* **121**, 9089-9095 (2017).
8. Patil, A. M., Lokhande, V. C., Lokhande, A. C., Chodankar, N. R., Ji, T., Kim, J. H. & Lokhande. C. D. Ultrathin nickel sulfide nano-flames as an electrode for high performance supercapacitor; comparison of symmetric FSS-SCs and electrochemical SCs device. *RSC Adv.* **6**, 68388-68401 (2016).
9. Liu,Y., Wang,W., Huang,H., Gu,L., Wang Y. & Peng, X. The highly enhanced performance of lamellar WS_2_ nanosheet electrodes upon intercalation of single-walled carbon nanotubes for supercapacitors and lithium ions batteries. *Chem. Commun.* **50**, 4485-4488 (2014).
10. Shang,X., Chi,J.Q., Lu,S.S., Gou,J.X., Dong,B., Li,X., Liu,Y.R., Yan,K.L., Chai,Y.M. & Liu, C.G. Carbon fiber cloth supported interwoven WS_2_ nanosplates with highly enhanced performances for supercapacitors. *Appl. Surf. Sci.* **392**, 708-714 (2017).
11. Hu,B., Qin,X., Asiri, A.M., Alamry,K.A., Al-Youbi, A.O. & Sun, X. WS_2_ nanoparticles-encapsulated amorphous carbon tubes: A novel electrode material for supercapacitors with a high rate capability. *Electrochem. Commun.* **28**, 75–78 (2013).
12. Fan,L.Q., Liu,G.J., Zhang,C.Y., Wu,J.H. & Wei,Y.L. Facile one-step hydrothermal preparation of molybdenum disulfide/carbon composite for use in supercapacitor. *Int. J. Hydrog. Energy* **40**, 10150-10157 (2015).
13. Song,Y., Chen,X.X., Zhang,J.X., Cheng,X.L. & Zhang,H.X. Freestanding micro-supercapacitor with interdigital electrodes for low-power electronic systems. *J. Microelectromech. S.* **26**, 1055-1062 (2017).
14. Bissett, M.A., Kinloch, I. A. & Dryfe, R.A. W. Characterization of MoS_2_-graphene composites for high-performance coin cell supercapacitors. *ACS Appl. Mater. Interfaces,* **7**, 17388–17398 (2015).
15. Xi,S., Zhu,Y., Yang,Y., Jiang,S. & Tang, Z. Facile synthesis of free-standing NiO/MnO_2_ core-shell nanoflakes on carbon cloth for flexible supercapacitors. *Nanoscale. Res. Lett.* **12**, 171 (2017).
16. Savjani, N., Lewis, E. A., Bissett, M. A., Brent, J. R., Dryfe, R. A.W., Haigh, S.J.& O'Brien, P. Synthesis of lateral size-controlled monolayer 1H-MoS_2_@Oleylamine as supercapacitor electrodes. *Chem. Mater.* **28***,* 657-664 (2016)*.*
17. Wang,S., Zhu,J., Shao,Y., Li,W., Wu,Y. & Hao,X. Three-dimensional MoS_2_@CNT/RGO network composites for high-Performance flexible supercapacitors. *Chem. Eur. J.* **23**, 3438-3446 (2017).
18. Balamuralitharan, B., Karthick, S. N., Balasingam, S. K., Hemalatha, K. V., Selvam, S., Raj, J.A. Prabakar, K. Jun,Y. & Kim, H.J. Hybrid reduced graphene oxide/manganese diselenide cubes: A new electrode material for supercapacitors. *Energy Technol.* **5**, 1953-1962 (2017).
19. Lia,D., Zhou,W., Zhou,Q., Yea, G., Wang, T., Wub, J., Chang,Y. & Xu.J. Transparent 1T-MoS_2_ nanofilm robustly anchored on substrate by layer-by-layer self-assembly and its ultra-high cycling stability as supercapacitors. *Nanotechnology* **28**, 395401(2017).
20. Tang, H., Wang, J., Yin,H., Zhao,H., Wang,D. & Tang,Z. Growth of polypyrrole ultrathin films on MoS_2_ monolayers as high-performance supercapacitor electrodes. *Adv. Mater.* **27**, 1117-1123 (2015).
21. Cao, L., Yang, S., Gao, W., Zheng L., Gong ,Y., Ma,L., Shi,G., Lei ,S., Zhang, Y., Zhang,S., Vajtai,R.; & Ajayan, P.M. Direct laser-patterned micro-supercapacitors from paintable MoS_2_ films. *Small* **9**, 2905-2910 (2013).
22. Soon, J. M., & Loh, K. P. Electrochemical double-layer capacitance of MoS_2_ nanowall films. *Electrochem. Solid-State Lett.***10**, A250-A254 (2007).

**Figure S1.**((a) XRD patterns and (b) Raman spectroscopy spectra of the as-grown ALD-SnS_x_ thin films on Nickel foam by 500 ALD cycles.
